# Estimating Population Immunity and Impact of COVID-19 Vaccination in Washington State and Oregon

**DOI:** 10.1093/ofid/ofaf531

**Published:** 2025-08-30

**Authors:** Mia Moore, Larissa Anderson, Chloe Bracis, David A Swan, Ian Painter, Erik Everson, Holly Janes, Joshua T Schiffer, Laura Matrajt, Dobromir Dimitrov

**Affiliations:** Fred Hutchinson Cancer Center, Seattle, Washington, USA; Fred Hutchinson Cancer Center, Seattle, Washington, USA; University of Grenobles Alpes, Grenoble, France; Fred Hutchinson Cancer Center, Seattle, Washington, USA; Washington State Department of Health, Shoreline, Washington, USA; Oregon Health Authority, Portland, Oregon, USA; Fred Hutchinson Cancer Center, Seattle, Washington, USA; Fred Hutchinson Cancer Center, Seattle, Washington, USA; Fred Hutchinson Cancer Center, Seattle, Washington, USA; Fred Hutchinson Cancer Center, Seattle, Washington, USA

**Keywords:** COVID-19, mRNA vaccines, counterfactual modeling, SARS-CoV-2 vaccine effectiveness, compartmental epidemic model

## Abstract

**Background:**

The severe acute respiratory syndrome coronavirus 2 (SARS-CoV-2) mRNA vaccine showed high clinical efficacy against the ancestral variant, but immunological waning, emergence of new variants, and the durability of infection-induced immunity complicate the estimation of population-level effectiveness. We used mathematical modeling to calculate the proportion of hospitalizations averted by vaccination in Washington and Oregon.

**Methods:**

We used an age- and region-structured compartmental model of vaccine-induced and infection-induced immunity from January 2020 until December 2022. We parameterized the strength and durability of immunity via a meta-regression of vaccine efficacy. We calibrated a time-varying contact matrix to weekly hospitalizations reported by the Washington Department of Health and Oregon Health Authority. We validated our model with Centers for Disease Control and Prevention serosurveillance data. To estimate vaccine effectiveness, we created counterfactual scenarios with no vaccination either in the entire population or in select age groups.

**Results:**

We found that total hospitalizations were reduced 74% (95% credible interval [CrI], 69%–78%) and 15% (95% CrI, 9%–19%) by primary vaccination and boosters, respectively. Vaccination effectiveness was highest during the Alpha wave, averting 90% (95% CrI, 88%–93%) of hospitalizations and in people aged 65+, averting 78% (95% CrI, 73%–81%). Relative to only vaccinating individuals aged 50+, vaccination of individuals aged 18–49 averted 52% (95% CrI, 44%–58%) of hospitalizations overall and 42% (95% CrI, 35%–48%) of hospitalizations among individuals 65+.

**Conclusions:**

The SARS-CoV-2 vaccination program in Washington and Oregon averted the majority of hospitalizations. Vaccinating individuals aged 18–49 significantly reduced hospitalization among individuals aged 65+.

The first death from coronavirus disease 2019 (COVID-19) in the United States was reported in March 2020 in Washington State. By March 2021, there were roughly 500 000 US deaths, with millions more hospitalized. However, a large-scale vaccination campaign was already underway in the United States with highly efficacious mRNA-based vaccines. To evaluate the real-world effectiveness of the mRNA vaccines, mathematical modelers have created counterfactual scenarios in which vaccination was completely or partly eliminated [1–5]. Two such studies estimated that, in the first year of the pandemic, the vaccine averted 79% of deaths globally and 50% in low- and middle-income countries [3, 5].

This task becomes more complex over time due to (1) the buildup of population-level immunity from infection, primary vaccination, and booster doses; (2) the spread of variants that can evade population immunity; and (3) the changing backdrop of nonpharmaceutical interventions as the epidemic waxes and wanes. Models incorporating the best current knowledge on vaccine efficacy over time, local vaccination rates, and local surveillance data have quantified the impact of vaccination on specific areas including Brazil, Japan, and Israel [1, 2, 4]. These studies highlighted the importance of rapid vaccination and the vaccination of younger age groups.

Here we quantify the impact of vaccines and booster doses in Washington and Oregon up to the end of 2022. We have previously quantified the durability of different types of immunity to both the pre-Omicron and Omicron variants in a systematic review and meta-regression [6]. Leveraging this information, we develop a detailed compartmental model of vaccine and infection-induced immunity to track population-level protection against infection and hospitalization. The model tracks vaccinated individuals with primary doses and boosters, primary infections, breakthrough infections, and reinfections. In addition, the model tracks waning immunity from vaccine- and naturally acquired immunity and hybrid immunity. Finally, the model separately tracks immunity to the pre-Omicron and Omicron variants. We then created counterfactual scenarios without (1) any vaccination, (2) vaccination of specific age groups, or (3) booster doses. We analyzed the impact of vaccination by projecting the proportion of hospitalizations averted under the true scenario, relative to our counterfactuals.

## METHODS

For the purpose of this paper, we consider severe disease to be any diagnosed case of COVID-19 resulting in hospitalization and/or death, as determined by the Washington and Oregon state health departments. To create vaccine-free counterfactual scenarios, we used a compartmental, age-structured epidemic model, with time-varying rates of transmission and vaccination. Our model incorporates (1) vaccine-induced and prior infection-induced protection from severe acute respiratory syndrome coronavirus 2 (SARS-CoV-2) breakthrough infection and COVID-19 hospitalization and (2) waning of that protection over time and due to strain replacement. We formulate and parameterize our model using our prior meta-analysis on the durability of immunity [6]. We reconstruct time-varying transmission rates using the number of weekly hospitalizations and infections by age and region in Washington State from January 1, 2020, to December 31, 2022, and Oregon from January 1, 2020, to August 31, 2022. We use these transmissions rates to simulate the number of hospitalizations and infections in the absence of vaccination.

### Compartmental Model

**Figure 1.**
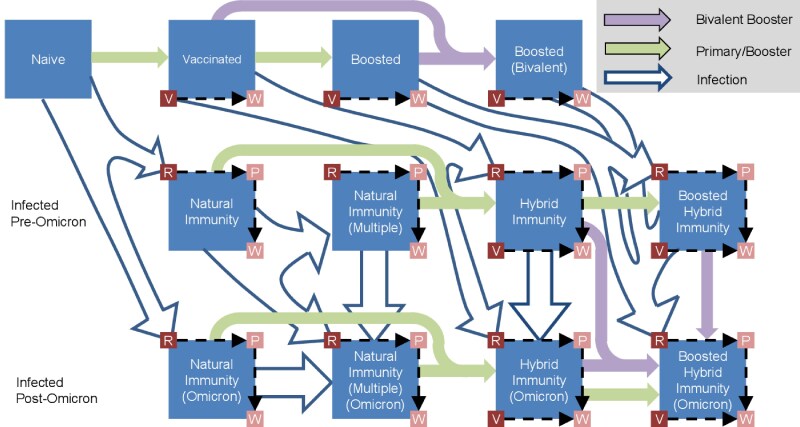
Immunity flowchart. All individuals start in the naive compartment and move between states based on vaccination, infection, or immune waning. Larger squares indicate classes, whereas small dark squares (“initial”) and small light squares (“waned”) squares show subclasses. Omicron-specific immunity is denoted by “Omicron/bivalent.” Arrows that loop back to the same state are omitted. For simplicity, all individuals with Omicron-specific immunity were assumed to also have pre-Omicron-specific immunity (but not vice versa). Abbreviations: P, waned immunity/anti-N-positive; R, recent infection; V, recent vaccination; W, waned immunity.

An individual's susceptibility to infection and severe disease depends on their history of immune-conferring events (ICEs), that is, vaccination or infection. We therefore subdivide the population into broad classes, indexed by *i*, based on the number and type of ICEs (Figure 1). Individuals in the “naive” class have had no ICEs at any point since the start of the pandemic and are the most susceptible to infection and disease. After vaccination or infection, individuals enter the “vaccinated” or “natural immunity” classes, respectively. Vaccinated individuals may receive a booster dose and become “boosted” or acquire an infection and gain “hybrid immunity.” Similarly, individuals with natural immunity may become reinfected (“natural immunity [multiple]”) or get vaccinated and gain hybrid immunity. Finally, individuals may gain “boosted hybrid immunity” following a primary vaccination, booster dose, and infection. Additional ICEs do not change the class of immunity. Each immunity class is further subdivided into subclasses, indexed by *k*, based on the amount of time since the most recent ICE (Figure 1; Supplementary Table 1).

Each class and subclass has an associated protection against infection and severe disease with variant *j*, denoted $C_{ij}^{k}$ and $M_{ij}^{k}$, respectively. We estimated these parameters using a systematic review and meta-regression of >100 studies in which we derived separate parameters for initial effectiveness and durability of infection- and vaccine-derived immunity (Supplementary Methods). This meta-analysis did not yield estimates for the bivalent booster immunity, which was assumed to provide equivalent protection against Omicron as the initial booster did against pre-Omicron or protection following reinfection, which was assumed to be equivalent to hybrid immunity. We also did not estimate separate values of these parameters for each age group.

Initially following an infection (subclass R) or vaccination (subclass V), *C* and *M* take the “initial” (high) values given in Supplementary Table 2. An average of 20 weeks following vaccination, individuals transition to subclass W, where immunity is reduced to “Waned” values. After a similar delay following an infection, individuals first transition to subclass P, where immunity is reduced but they remain seropositive for antinucleocapsid antibodies. Over a longer time, individuals in the P subclass become seronegative and transition to the W subclass. We parameterized the rate of seroreversion based on analysis of the Roche-N and Abbott-N assays (Supplementary Figure 1) [7]. This extended period of seropositivity has no impact on immunity but allows for additional model validation with serosurveillance data (Supplementary Figure 2). Further details are available in the Supplementary Methods.

We distinguish between “pre-Omicron immunity” conferred by primary vaccination, the original boosters, or infection with a pre-Omicron variant and “Omicron immunity” conferred by infection with an Omicron variant or vaccination with the bivalent booster (which contained both pre-Omicron and Omicron variants). Once an individual has acquired Omicron immunity, they retain it for the rest of the simulation; however, the protection that it affords them will still wax and wane depending on the timing of immune-conferring events. We separately parameterize immune protection against “matched” and “unmatched” variants (Supplementary Table 2). Individuals with pre-Omicron immunity have “matched” immunity only against pre-Omicron variants, while individuals with Omicron-specific immunity have “matched” immunity against all variants. This latter assumption simplifies our model by leveraging the fact that very few individuals would have exposure to pre-Omicron variants following an Omicron infection.

We define the population-level immunity as the reduction in either infection (*EC*) or severe disease (*EM*) relative to a naive population. These values can be calculated from the model by averaging *C* and *M* across the entire population weighted by the number of individuals in each class and subclass (Supplementary Methods).

### Infection, Vaccination, and Transmission

We define the *force of infection* ($\lambda_{\;j,ar}^{T}$) as the daily risk of infection from variant *j* at time *t* in age group *a* and for region *r*. The *force of vaccination* ($\nu_{\;j,ar}^{T}$) is the per-capita rate of vaccination for vaccine *j* at time *t* among those eligible for vaccination (ie, those who have not recently received a vaccination). We calibrate $\lambda_{\;j,ar}^{T}$ and $\nu_{\;j,ar}^{T}$ for each variant (Ancestral, Alpha, Delta, and Omicron) and force of vaccination for each vaccine (primary series, booster, and bivalent booster) using weekly hospitalization and vaccination data in Washington State and Oregon, provided by the respective state departments of health, as well as data from the Centers for Disease Control and Prevention on the distribution of variants over time (Supplementary Figures 3–6). For more details, see the Supplementary Methods.

Pharmaceutical interventions such as vaccination and nonpharmaceutical interventions such as social distancing and masking have both direct and indirect effects on viral transmission. We define direct effects as the decreased risk of infection and severe disease given a constant force of infection (changes in *C* and *M*), whereas the indirect effects are the reduction in the future force of infection over time (changes in $\lambda_{\;j,ar}^{T}$). To quantify indirect effects, we estimate the number of secondary infections that a single index case of variant j in age group $a^{\prime }$, will lead to in age group *a* in the subsequent week. Within our model, transmission is represented as a time-varying matrix $B_{\;j,r}^{T}$, which gives the number of new infections in each age group from 1 week to the next in a hypothetical, completely susceptible population (Supplementary Figure 7). The leading eigenvalue of this matrix, $B_{\;j,r}^{T}$, is the exponential growth rate in a completely susceptible population or roughly $R_{0}$ divided by the generation time in weeks. We label this quantity the *efficacy adjusted weekly growth rate*. For more details, see the Supplementary Methods.

To create a counterfactual scenario with no vaccination, we resimulated our model with $\nu_{\;j,ar}^{T}=0$. We assume that the force of infection from outside the population, $\lambda_{\;j,ar,0}^{T}$ (Supplementary Figure 8), remains the same across scenarios with variants being introduced at the same time. Similarly, we assume that all other nonpharmaceutical interventions and contact behaviors remain unchanged, so $B_{\;j,r}^{T}$ is unchanged across all scenarios. In addition, we consider additional scenarios where vaccination is only removed for age group $\tilde{a}$, that is, $\nu_{\;j,\tilde{a}r}^{T}=0$, and in which there are no booster doses, that is, $\nu_{\;j=\mathrm{boosters},ar}^{T}=\nu_{\;j=\mathrm{bivalent}\;\mathrm{boosters},ar}^{T}=0$.

## RESULTS

### COVID Immunity Over Time Across Washington and Oregon

**Figure 2.**
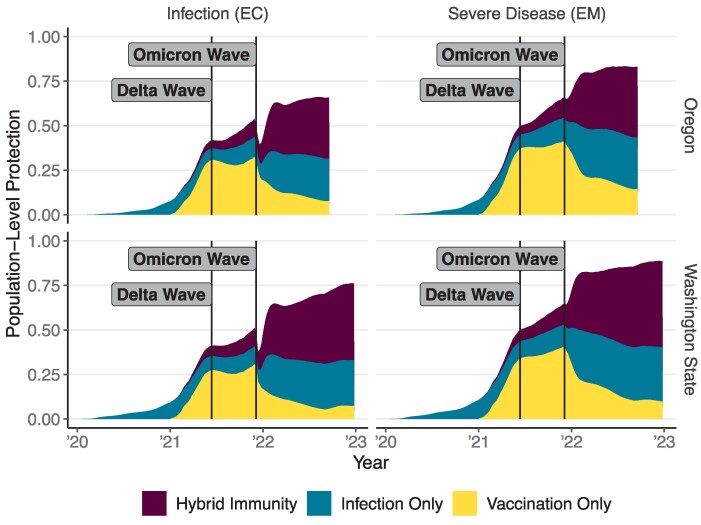
Population-level immunity to SARS-CoV-2 infection (EC) and severe COVID-19 (EM) over time in Washington and Oregon State. The total height of the shaded area corresponds to the reduction in incidence of each outcome relative to a naive population. Individual shaded areas show the contribution of individuals with vaccine-induced, infection-induced, and hybrid immunity. Abbreviations: COVID-19, coronavirus disease 2019; SARS-CoV-2, severe acute respiratory syndrome coronavirus 2.

We first calculate the population-level immunity over the first 3 years of the COVID pandemic (Figure 2). This population-level immunity is defined as the reduction in infections and severe disease, based on the variants circulating at a given time. We estimate that by the end of 2020, infection-induced immunity was sufficient to reduce infections by 9.0% (95% credible interval [CrI], 6.8%–12.5%) and 7.2% (95% CrI, 5.7%–8.9%) in Washington State and Oregon, respectively, and to reduce severe outcomes by 46.2% (95% CrI, 14.0%–75.8%) and 30.9% (95% CrI, 6.9%–61.5%) in Washington State and Oregon, respectively. These results are similar to those obtained by the COVIDestim team during this time frame [8]. By the start of the Delta wave in June of 2021, vaccination had increased overall population immunity to infection to 41.4% (95% CrI, 38.7%–45.2%) and 41.9% (95% CrI, 39.4%–44.4%) in Washington State and Oregon, respectively. Immunity to severe disease also increased to 77.9% (95% CrI, 62.8%–90.0%) and 71.2% (95% CrI, 60.9%–84.4%) in Washington State and Oregon, respectively. Throughout the fall of 2021, booster doses further increased immunity to infection to 51.6% (95% CrI, 48.1%–57.0%) and 54.3% (95% CrI, 49.9%–57.7%) in Washington State and Oregon, respectively. Immunity to infection dipped briefly following the emergence of Omicron, reflecting our assumption that pre-Omicron-specific immunity conferred less protection against Omicron. By September 2022, immunity to infection was 71.8% (95% CrI, 61.2%–80.8%) and 67.0% (95% CrI, 57.1%–74.8%) in Washington State and Oregon, respectively. Immunity to severe disease at this time point was 87.6% (95% CrI, 77.0%–95.5%) and 82.9% (95% CrI, 73.9%–91.4%) in Washington State and Oregon, respectively. These numbers, as well as the breakdown by type of immunity, were also similar to what was found by the COVIDestim team [9]. See Supplementary Figures 9 and 10 for breakdowns by age and region.

### Estimation of Weekly Transmission and Infection Rates

We estimated the weekly growth rate in infections in a completely naive population. Initially, this number was between 2 and 3 in both Washington State and Oregon, but it dropped to close to 1 in the early part of 2020 due to nonpharmaceutical interventions such as social distancing. During 2021 and 2022, the weekly growth rate gradually increased to around 6, but population immunity to infection during that period was sufficient to keep the infection from rapidly spreading (Supplementary Figure 11). This increase could be driven by either higher $R_{0}$ for the circulating strains or increased contact rates.

We simulated a counterfactual COVID epidemic scenario with no vaccination in Washington State and Oregon and compared the weekly number of infections with those seen with the true vaccination rate (Supplementary Figure 12). In both the counterfactual and actual scenarios, there were 2 very large peaks of infection—the Delta wave in the summer of 2021 and the Omicron wave in winter 2021–2022. The Delta wave was significantly larger and peaked earlier in the counterfactual scenario. In contrast, both scenarios had similar numbers of infections during the Omicron wave.

### Majority of Severe Outcomes Averted by Vaccination

**Figure 3.**
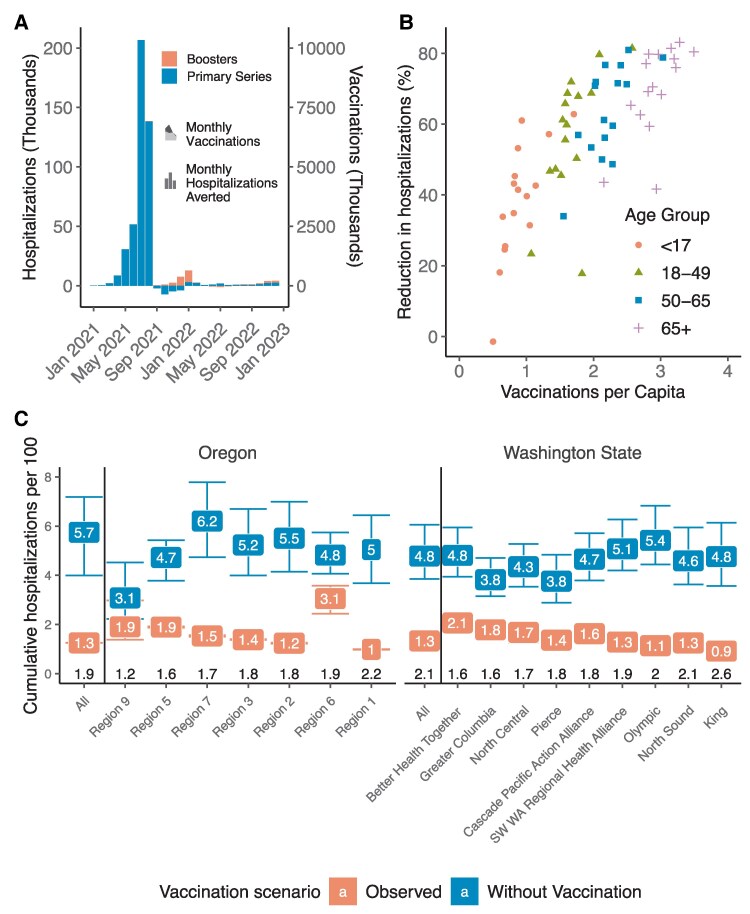
Estimated impact of vaccination on hospitalization in Washington State and Oregon. A, Monthly impact of vaccines. Bars indicate the difference in the median number of hospitalizations by month between simulation with primary vaccination only and no vaccination (blue) and between simulations with booster and primary vaccination only (red). Lightly shaded areas show the monthly numbers of each type of vaccination. B, Correspondence of vaccine uptake (in doses per person) and impact (as percent reduction in hospitalizations in simulation with vaccination vs without). Points represent median projections across all simulations for a single age group in the region of either Washington State or Oregon. C, Per-capita hospitalization rates over the duration of the simulation in each region of Washington State and Oregon, arranged by vaccine uptake. White labels show cumulative incidence of hospitalization. Black label indicates number of vaccine doses per person.

The rate of severe outcomes is calculated from infection rates using the variant- and age-specific severity rates in each immunity class and subclass. In the absence of vaccination and with no other changes in behavior, we projected that there would have been an additional 444.9K (95% CrI, 323.1K–555.9K) hospitalizations combined in Washington State and Oregon (Table 1) from 2020 to 2022. Vaccination therefore averted 74.3% (95% CrI, 68.5%–78.3%) of hospitalizations during this period according to our model. It specifically averted 90.4% (95% CrI, 87.8%–92.6%) and 87.0% (95% CrI, 81.9%–89.8%) of hospitalizations during the Alpha and Delta waves (Figure 3*A*). Not surprisingly, individuals 65 and over benefitted the most from vaccination, with 256.0K (95% CrI, 195.2K–306.7K) hospitalizations averted, a reduction of 77.5% (95% CrI, 72.8%–80.5%). There was a wide disparity in the per-capita vaccine uptake, ranging from 1.1 doses per capita in Eastern Oregon's rural Region 9 to 2.6 per capita in Washington's King region, which encompasses the entire Seattle metropolitan area. Across regions and age groups, our model projected a strong relationship between uptake of vaccines and proportion of infections averted (Figure 3*B* and *C*).

**Table 1.** Simulated Numbers of Hospitalizations (in Thousands) With Vaccination Levels (at Observed Levels), No Vaccination, or Primary Vaccination Only (No Booster Doses)

	Hospitalization (Thousands)			Reduction (%) From	
	With Vaccination	No Vaccination	No Boosters	Vaccination	Boosters
Age group					
0–17 y	4 (4–4)	8 (7–9)	4 (4–5)	50.1 (40.4–57.3)	6.9 (1.5–11.6)
18–49 y	39 (39–39)	131 (95–159)	43 (41–44)	70.4 (60.0–75.4)	8.5 (5.2–11.7)
50–64 y	37 (36–37)	132 (104–165)	42 (40–43)	72.2 (65.2–77.7)	11.8 (7.9–15.5)
65+ y	74 (74–75)	330 (270–381)	92 (85–99)	77.5 (72.8–80.5)	19.4 (13.0–24.5)
Variant					
Ancestral	44 (44–44)	45 (45–46)	44 (44–44)	3.1 (2.5–3.5)	0.0 (0.0–0.0)
Alpha	10 (10–10)	106 (82–136)	10 (10–10)	90.4 (87.8–92.6)	0.5 (0.4–0.6)
Delta	50 (49–50)	383 (267–486)	60 (58–63)	87.0 (81.9–89.8)	17.6 (14.0–21.2)
Omicron	51 (50–51)	70 (54–87)	67 (57–77)	27.4 (6.5–41.9)	24.0 (11.8–34.0)
Oregon	53 (53–53)	242 (168–303)	61 (59–64)	78.2 (68.7–82.5)	13.7 (9.7–17.5)
Region 1	19 (19–20)	99 (72–126)	24 (22–25)	80.5 (73.0–84.7)	18.7 (13.4–23.6)
Region 2	10 (10–10)	45 (34–57)	12 (11–13)	77.5 (69.8–82.2)	14.3 (8.5–19.2)
Region 3	8 (8–8)	30 (23–39)	9 (9–10)	73.4 (65.4–79.3)	13.0 (8.6–17.2)
Region 5	6 (6–6)	15 (12–17)	7 (6–7)	59.9 (50.1–65.1)	10.0 (6.9–12.8)
Region 6	2 (1–2)	3 (2–3)	2 (1–2)	38.2 (30.0–44.2)	3.8 (−0.2–8.2)
Region 7	5 (5–5)	21 (16–26)	6 (6–6)	75.3 (67.2–80.6)	13.4 (8.8–19.4)
Region 9	3 (2–5)	5 (4–8)	4 (3–5)	37.4 (27.6–46.8)	7.1 (2.5–11.9)
Washington	101 (101–102)	364 (293–461)	120 (109–130)	72.2 (65.2–78.0)	15.1 (6.7–21.9)
Better Health Together	13 (13–13)	30 (24–37)	14 (13–15)	56.8 (47.6–65.2)	7.7 (1.7–13.2)
Cascade Pacific Action Alliance	11 (10–11)	30 (25–37)	12 (11–13)	65.1 (56.7–71.7)	12.9 (4.3–20.3)
Greater Columbia	13 (13–13)	28 (23–35)	14 (14–15)	53.1 (42.9–62.1)	7.7 (3.1–12.5)
King	21 (20–21)	107 (80–138)	27 (24–30)	80.8 (74.2–85.0)	23.1 (12.0–31.1)
North Central	4 (3–4)	11 (9–14)	5 (4–5)	62.2 (52.3–71.7)	8.8 (4.5–12.4)
North Sound	16 (16–16)	59 (47–76)	19 (17–22)	72.7 (65.4–78.9)	17.5 (7.9–25.3)
Olympic	4 (4–4)	21 (17–26)	5 (5–6)	79.0 (74.2–83.2)	18.3 (7.8–25.0)
Pierce	12 (12–13)	34 (26–44)	14 (13–15)	63.7 (52.9–71.2)	12.3 (7.8–17.9)
SW WA Regional Health Alliance	7 (7–7)	27 (22–33)	8 (8–9)	74.6 (69.2–79.7)	20.5 (13.0–28.0)
Combined	154 (154–155)	599 (478–710)	180 (170–191)	74.3 (68.5–78.3)	14.6 (9.4–19.1)

Percent reduction from vaccinations and boosters were calculated by comparing hospitalizations with vaccinations with a scenario with no vaccinations or boosters. Parenthetical ranges are 95% credible intervals.

Booster doses, which became available toward the end of the Delta wave, primarily affected transmission during the Omicron wave. Uptake was more limited, with only 38%, 7%, and 14% receiving third, fourth, and bivalent doses, respectively, compared with 68% receiving the second dose. Despite this, we projected that boosting averted 27.4% (95% CrI, 6.5%–41.9%) of Omicron hospitalizations overall and 40.5% (95% CrI, 23.6%–53.1%) among individuals 65+. Again, there was a strong correlation between the uptake of vaccines by a population and the proportion of infections averted across regions and age groups (Supplementary Figure 13).

### Vaccinating Younger Age Groups Protected Older Age Groups

To investigate the role of direct vs indirect effects of vaccination, we also simulated counterfactuals in which there was no vaccination of a specific age group (Table 2), but vaccination was administered at the observed level in the remaining age groups.

**Table 2.** Absolute (Thousands) and Relative (%) Numbers of Hospitalizations Averted From Vaccinating Specific Age Groups

	Vaccinated Age Group (Size of Age Group in Millions)					
	18–49 y (5.0)		50–64 y (2.2)		65+ y (2.0)	
Age Group	Hosps. Aver. (K)	Rel. Diff. (%)	Hosps. Aver. (K)	Rel. Diff. (%)	Hosps. Aver. (K)	Rel. Diff. (%)
18–49 y	76.4 (54.9–98.0)	66.2 (58.8–71.5)	18.9 (7.1–31.1)	33.0 (15.2–44.9)	6.9 (2.8–15.4)	15.2 (6.8–28.2)
50–64 y	32.2 (21.6–45.0)	46.7 (37.4–55.2)	54.4 (39.6–71.2)	59.9 (52.2–66.0)	6.4 (2.9–14.4)	14.7 (7.5–28.2)
65+ y	54.8 (38.3–68.7)	42.4 (34.6–48.0)	27.7 (11.3–42.5)	27.3 (13.2–37.0)	114.6 (95.0–147.3)	60.7 (56.3–66.5)
All ages	167.3 (116.2–214.5)	52.0 (43.6–58.2)	104.0 (57.9–148.1)	40.4 (27.6–49.0)	129.2 (101.3–176.9)	45.5 (40.0–53.3)

Hospitalizations averted is defined as the difference in number of hospitalizations between (1) vaccinating all individuals at the observed rates of vaccination and (2) setting the vaccination rate in 1 age group to 0 while vaccinating all others at observed rates. Columns correspond to the age group whose vaccinations have been removed, rows to the hospitalized age group(s). The range is the 95% credible interval.

As an example, we considered a counterfactual scenario in which no vaccines had been provided to individuals aged 65+ but that vaccination had continued as observed in the other age groups. Using this counterfactual as a baseline, we found that the marginal benefit of vaccinating individuals aged 65+ was 129.2K (95% CrI, 101.3K–176.9K) hospitalizations averted overall, of which 114.6K (95% CrI, 95.0K–147.3K) were in the 65+ age group. Thus, providing vaccination to individuals aged 65+ reduced hospitalizations by 45.5% (95% CrI, 40.0%–53.3%) overall and by 60.7% (95% CrI, 56.3%–66.5%) among this age group.

We created similar counterfactuals with no vaccination of individuals aged 50–64 and 18–49, respectively. We found that the vaccinating individuals aged 50–64 averted 104.0K (95% CrI, 57.9K–148.1K) hospitalizations, and only half, 54.4K (95% CrI, 39.6K–71.2K), were in the same age group. Vaccinating individuals aged 18–49 prevented the most hospitalizations, 167.3K (95% CrI, 116.2K–214.5K), including 32.2K (95% CrI, 21.6K–45.0K) among 50–64-year-olds and 54.8K (95% CrI, 38.3K–68.7K) among those age 65+. Vaccinating younger age groups thus provided a substantial benefit to older age groups due to the prevention of secondary transmission.

## DISCUSSION

The rapid development of mRNA-based vaccines against SARS-CoV-2 proved to be one of the most effective responses to the COVID-19 pandemic. In this counterfactual analysis of the epidemic in Washington State and Oregon, we show that even accounting for the natural immunity afforded by prior infection, vaccination reduced the numbers of severe infections dramatically during 2021 and 2022. This reduction was particularly large during the Alpha and Delta waves of 2021, when we estimated 90% and 87% fewer hospitalizations, respectively.

COVID-19 disease is more severe in older individuals, with both the case:hospitalization ratio and hospitalization:fatality ratio increasing with age [10]. For this reason, most jurisdictions prioritized vaccination based on age, resulting in greater vaccine uptake by older individuals. Consequently, individuals 65 and over benefited the most from the vaccine program both in absolute and relative terms. However, we also found that vaccination of individuals aged 18–49 had large direct and indirect effects, benefiting both themselves and older individuals.

As the epidemic progressed, many individuals acquired hybrid immunity, which can provide robust and effective protection against both infection and severe disease [11, 12]. However, booster doses also had a substantial impact on the number of hospitalizations. In particular, our analysis suggests that booster doses averted 24% of hospitalizations due to the Omicron variant. This decrease was substantially greater in subpopulations with more uptake, reaching as high as 40%. This highlights the importance of continued boosting doses even in highly immune populations.

This work had a number of limitations. As our intent was to focus on the impact of the vaccine, we assumed that all other nonpharmaceutical interventions, such as social distancing, remained unchanged in our counterfactual scenarios. The relationship between COVID-19 epidemic dynamics, risk perception, and social distancing behavior is complex, but individuals and governments would likely have re-implemented distancing protocols when faced with the Delta wave in the absence of the vaccine [13–15]. The consequences of these decisions are beyond the scope of this model. In addition, we have not modeled the strain on the health care system that such an enormous increase in hospital admissions would entail, or the consequences for COVID outcomes [16]. Second, to simplify the model, we assumed that an Omicron infection conferred protection equally against both Omicron and pre-Omicron variants, likely overestimating protection against the latter. This assumption would lead to a slight overestimate of strength of natural immunity in late 2021. However, the duration of Delta and Omicron overlap was small and decreased further in the counterfactual scenario, suggesting that the impact was likely quite small. Third, we did not account for population heterogeneity in vaccine efficacy or susceptibility to COVID infection, outside of age-specific contact rates. Lower vaccine efficacy among more vulnerable populations, such as those with comorbidities, would likely further highlight the importance of large-scale vaccination. Fourth, we used a combined end point of hospitalization and/or death as a proxy for severe COVID. However, many hospitalized cases, particularly those with the Omicron variant, may not have been due to COVID infection, potentially inflating estimates of severe cases and thus infection numbers. We note that (1) our model validation, based on seropositivity data, does not suggest a systematic overestimation of infections, but serosurveillance became less widespread in 2022; and (2) such a bias would primarily affect the absolute rather than relative impact of vaccination.

The vaccination program in Washington and Oregon had a considerable positive impact on the number of COVID hospitalizations. The indirect benefits of vaccination, particularly when vaccinating younger individuals, played a large role in improving the campaign's effectiveness. Even accounting for the robustness of natural immunity, we found that booster doses also led to a substantial reduction in hospitalization numbers, especially in regions with high uptake. Age-based restrictions on booster doses will significantly increase population-wide transmission rates, leading to greater numbers of hospitalizations even among older age groups.

## Supplementary Material

ofaf531_Supplementary_Data

## Supplementary Data

Supplementary materials are available at *Open Forum Infectious Diseases* online. Consisting of data provided by the authors to benefit the reader, the posted materials are not copyedited and are the sole responsibility of the authors, so questions or comments should be addressed to the corresponding author.
