# Supplementary material for: Estimating Population Immunity and Impact of COVID-19 Vaccination in Washington State and Oregon
Source: Open Forum Infect Dis. 2025 Aug 30;12(9):ofaf531. doi: 10.1093/ofid/ofaf531 (PMC12456170; doi:10.1093/ofid/ofaf531)
Supplement: ofaf531_Supplementary_Data [file ofaf531_supplementary_data.docx]

# Supplement: Estimating population immunity and impact of COVID-19 vaccination in Washington State and Oregon

## Supplemental Methods

### Compartmental Model

For each age group and region, we use a compartmental model to track the number of individuals, $X_{i,ar}^{k}$, in class $i$, where $k=\{V,R,P,W\}$ denotes the subclass (Supplementary Table S1). Individuals transition between immune classes following vaccination, infection, or the waning of immunity. The rates of vaccination and infection depend on time-varying “force” parameters. The force of vaccination, $\nu_{j,ar}^{T}$, is the daily per-capita rate of vaccination among those eligible for vaccination during week $T$ where $j\in\{\text{pri},\text{bst},\text{bvb}\}$ indicates primary vaccination, booster, and bivalent boosters. Similarly, the force of infection, $\lambda_{j,ar}^{T}$, represents the per-capita daily infection rate of the naive population in week $T$, where $j\in\{\text{anc},\text{alp},\text{del},\text{omi}\}$, indicate the ancestral, alpha, delta, and omicron variants. Eligibility of an individual in a specific class $i$ and subclass $k$, $E_{ij}^{k}$, depends on an individual’s vaccination history. For example, previously vaccinated people cannot get the primary series again, unvaccinated individuals cannot receive boosters before the primary vaccine, and individuals whose vaccine-induced immunity has not yet waned are not eligible for vaccination. Infection rates for specific classes and subclasses depend on their susceptibility to infection which is reduced for those with vaccine-induced or natural immunity by a factor $C_{ij}^{k}$ (Supplementary Table S2). The proportion of individuals with severe infection, $M_{ij}^{k}$, depends on pre-existing immunity as well as variant severity $\mu_{j,a}$. Variant severity varies by age group and variant where $\frac{\mu_{j,a}}{1-\mu_{j,a}}=\text{OR}_{\text{SEV},j}\frac{\mu_{\text{anc}\text{,a}}}{1-\mu_{\text{anc}\text{,a}}}$ (Supplementary Table S3). After vaccination and infection, individuals enter a new class, determined by the transition matrices $Y^{V}$ and $Y^{I}$ (Supplementary Table S4), respectively. Waning of immunity occurs continuously via a transition from a more protected subclass ($k=R$ or $k=V$) to a less protective one, at rate $\omega_{k}$. Forcing parameters $\nu_{j,ar}^{T}$ and $\lambda_{j,ar}^{T}$ are calibrated by computing the total number of vaccinations and severe infections by week, $Z_{j,ar}^{T}$, and comparing to observation (see [estimation of λ and ν](#_Estimation_of_𝜆) and Supplementary Figures S2 – S6). This process is repeated for each group and geographic region, with only the rates of symptomatic and severe infection changing between age groups. Supplementary Table S5 summarizes the parameters used in the simulation. Note that in the equations below, the subscripts “ar” have been suppressed for clarity.

$$\begin{matrix} \begin{matrix} \text{Naive} \\ k=⌀, i=0, t\in[t_{T},t_{T+1}) \end{matrix} \frac{dX_{0}}{dt} & =-\underset{\text{Infection}}{\underset{⏟}{\sum_{j\in\{\text{anc},\text{alp},\text{del},\text{omi}\}} \lambda_{j}^{T}X_{0}}}-\underset{\text{Primary vaccination}}{\underset{⏟}{\nu_{\text{pri}}^{T}X_{0}}} \\ \begin{matrix} \text{Recent Vaccination Only} \\ k=V, i>0\mathbb{\in I,} t\in[t_{T},t_{T+1}) \end{matrix} \frac{dX_{i}^{V}}{dt} & =\underset{\text{Waning of vaccine-induced immunity}}{\underset{⏟}{-\omega_{V}X_{i}^{V}}}-\underset{\text{Breakthrough infection}}{\underset{⏟}{\sum_{j\in\{\text{anc},\text{alp},\text{del},\text{omi}\}} \lambda_{j}^{T}C_{ij}^{V}X_{i}^{V}}} \\ & +\underset{\text{Vaccination}}{\underset{⏟}{\sum_{j\in\text{pri},\text{bst},\text{bvb}} \nu_{j}^{T}\sum_{i\mathbb{'\in I}} Y_{i'\to i,j}^{V}E_{i'j}^{W}X_{i'}^{W}}} \\ \begin{matrix} \text{Recent Infection} \\ k=R, i>0\mathbb{\in I,} t\in[t_{T},t_{T+1}) \end{matrix}\frac{dX_{i}^{R}}{dt} & =\underset{\text{Waning of natural immunity}}{\underset{⏟}{-\omega_{R}X_{i}^{R}}}+\underset{\text{Vaccination of recently infected}}{\underset{⏟}{\sum_{j\in\text{pri},\text{bst},\text{bvb}} \nu_{j}^{T}\sum_{i\mathbb{'\in I}} Y_{i'\to i,j}^{V}E_{i'j}^{R}X_{i'}^{R}}} \\ & -\underset{\text{Re-infection}}{\underset{⏟}{\sum_{j\in\{\text{anc},\text{alp},\text{del},\text{omi}\}} \lambda_{j}^{T}C_{ij}^{R}X_{i}^{R}}}-\underset{\text{Vaccination}}{\underset{⏟}{\sum_{j\in\{\text{pri},\text{bst},\text{bvb}\}} \nu_{j}^{T}E_{ij}^{R}X_{i}^{R}}} \\ & +\underset{\text{Infection}}{\underset{⏟}{\sum_{j\in\text{anc},\text{alp},\text{del},\text{omi}} \lambda_{j}^{T}\sum_{i\mathbb{'\in I}} Y_{i'\to i,j}^{I}\sum_{k\in\{V,R,P,W\}} C_{i'j}^{k}X_{i'}^{k}}} \\ \begin{matrix} \text{Sero}\text{p}\text{ositive (Nucleocapsid)} \\ k=P, i>0\mathbb{\in I,} t\in[t_{T},t_{T+1}) \end{matrix}\frac{dX_{i}^{P}}{dt} & =\omega_{R}X_{i}^{R}\underset{\text{Seroreversion}}{\underset{⏟}{-\omega_{P}X_{i}^{P}}}+\underset{\text{Vaccination of seropositive}}{\underset{⏟}{\sum_{j\in\text{pri},\text{bst},\text{bvb}} \nu_{j}^{T}\sum_{i\mathbb{'\in I}} Y_{i'\to i,j}^{V}E_{i'j}^{P}X_{i'}^{P}}} \\ & -\underset{\text{Re-infection}}{\underset{⏟}{\sum_{j\in\{\text{anc},\text{alp},\text{del},\text{omi}\}} \lambda_{j}^{T}C_{i'j}^{P}X_{i'}^{P}}}-\underset{\text{Vaccination}}{\underset{⏟}{\sum_{j\in\{\text{pri},\text{bst},\text{bvb}\}} \nu_{i}^{T}E_{i'j}^{P}X_{i'}^{P}}} \\ \begin{matrix} \text{Waned Immunity} \\ k=W, i>0\mathbb{\in I,} t\in[t_{T},t_{T+1}) \end{matrix}\frac{dX_{i}^{W}}{dt} & =\omega_{V}X_{i}^{V}+\omega_{P}X_{i}^{P}-\underset{\text{Re-infection}}{\underset{⏟}{\sum_{j\in\{\text{anc},\text{alp},\text{del},\text{omi}\}} \lambda_{ij}^{T}C_{ij}^{W}X_{i}^{W}}} \\ & -\underset{\text{Vaccination}}{\underset{⏟}{\sum_{j\in\{\text{pri},\text{bst},\text{bvb}\}} \nu_{j}^{T}E_{ij}^{W}X_{i}^{W}}} \\ \begin{matrix} \text{Total Vaccinations} \\ j\in\{\text{pri},\text{bst},\text{bvb}\},\forall T \end{matrix} Z_{j}^{T} & =\nu_{j}^{T}\int_{t_{T}}^{t_{T+1}} \sum_{i} \sum_{k\in\{S,P,W\}} E_{ij}^{k}X_{i}^{k}\left( t \right) dt \\ \begin{matrix} \text{Severe Infections} \\ j\in\{\text{anc},\text{alp},\text{del},\text{omi}\},\forall T \end{matrix} Z_{j}^{T} & =\lambda_{j}^{T}\int_{t_{T}}^{t_{T+1}} \sum_{i} \sum_{k\in\{V,R,P,W\}} C_{ij}^{k}M_{ij}^{k}X_{i}^{k}\left( t \right) dt \end{matrix}$$

Supplementary Table S1: Summary of subclasses and their relationships. 'Ab' indicates whether individuals in the subclass are seropositive for anti-nucleocapsid antibody. 'Immunity' indicates which level of protection is used. 'Waning', 'Vaccination', and 'Infection' indicate the subclasses individuals go to following each transition. Note that individuals in the V class cannot be vaccinated again as there would be insufficient time between vaccinations. The modeled vaccines target only the spike protein and do not generate anti-nucleocapsid antibodies.

| Sub-class | Description | Ab | Immunity | Waning | Vaccination | Infection |
| --- | --- | --- | --- | --- | --- | --- |
| $V$ | Recent vaccination | - | Initial | $\to W$ | $\text{None}$ | $\to R$ (in new class) |
| $R$ | Recent infection | + | Initial | $\to P$ | $\to R$ (in new class) | $\to R$ (in new class) |
| $P$ | No recent ICE | + | Waned | $\to W$ | $\to R$ (in new class) | $\to R$ (in new class) |
| $W$ | No recent ICE | - | Waned | None | $\to V$ (in new class) | $\to R$ (in new class) |

Supplementary Table S2: Efficacy parameters. Reduction in susceptibility to infection (1 - $C_{ij}^{k}$) and severe outcomes (1 - $M_{ij}^{k}$) due to immunity induced by mRNA-based vaccination and/or prior infection. Values were estimated from systematic literature review and metaregression. Immediately following the immune conferring event, an individual benefits from the 'Initial' value, but their immunity is reduced to the 'Waned' value over time. If the infecting variant is the same as the variant from the prior infection (or used to formulate the vaccine), they benefit from the 'matched' protection. Values in each iteration were sampled uniformly from the ranges, which represent the 95% credible intervals from our analysis. Where multiple entries have the same range, a single value was used for each iteration.

|  | | Matched | | Not Matched | |
| --- | --- | --- | --- | --- | --- |
| Outcome | Immune Conferring Event(s) | Initial (V/S) | Waned (P/W) | Initial (V/S) | Waned (P/W) |
| Any Infection | Vaccination (Primary Series) | 88.4 (86.4 - 90.4) | 41.8 (33.9 - 49.7) | 49.5 (43.4 - 55.7) | -17.7 (-30.5 - -5.0) |
|  | Infection | 88.4 (86.4 - 90.4) | 75.5 (64.5 - 86.6) | 46.5 (36.1 - 56.8) | 22.0 (1.1 - 42.9) |
|  | Vaccination + Infection | 88.4 (86.4 - 90.4) | 75.5 (64.5 - 86.6) | 77.1 (65.9 - 88.3) | 32.1 (9.8 - 54.3) |
|  | Vaccination (Booster) | 88.4 (86.4 - 90.4) | 75.5 (64.5 - 86.6) | 64.9 (58.5 - 71.3) | 10.1 (-5.8 - 26.0) |
|  | Booster + Infection | 88.4 (86.4 - 90.4) | 75.5 (64.5 - 86.6) | 87.3 (75.3 - 99.2) | 32.1 (9.8 - 54.3) |
|  | Two+ Infections | 88.4 (86.4 - 90.4) | 75.5 (64.5 - 86.6) | 87.3 (75.3 - 99.2) | 32.1 (9.8 - 54.3) |
| Severe Disease | Vaccination (Primary Series) | 93.6 (91.6 - 95.5) | 82.2 (77.9 - 86.5) | 77.0 (72.7 - 81.3) | 66.8 (57.4 - 76.3) |
|  | Infection | 93.6 (91.6 - 95.5) | 82.2 (77.9 - 86.5) | 92.0 (88.5 - 95.6) | 81.9 (71.3 - 92.4) |
|  | Vaccination + Infection | 93.6 (91.6 - 95.5) | 82.2 (77.9 - 86.5) | 92.0 (88.5 - 95.6) | 81.9 (71.3 - 92.4) |
|  | Vaccination (Booster) | 93.6 (91.6 - 95.5) | 82.2 (77.9 - 86.5) | 92.0 (88.5 - 95.6) | 73.3 (63.8 - 82.8) |
|  | Booster + Infection | 93.6 (91.6 - 95.5) | 82.2 (77.9 - 86.5) | 92.0 (88.5 - 95.6) | 81.9 (71.3 - 92.4) |
|  | Two+ Infections | 93.6 (91.6 - 95.5) | 82.2 (77.9 - 86.5) | 92.0 (88.5 - 95.6) | 81.9 (71.3 - 92.4) |

Supplementary Table S3: Literature values for each variant for generation time, the upper bound of $R_{0}$, odds ratio of symptoms, and odds ratio of severe symptoms. Generation times were equated to incubation rates.(Wu et al. 2022) We used upper bounds of $R_{0}$ from a systematic review and meta-analysis, except for that of the Ancestral Variant which we back-calculated from an estimate of the increased transmissability of Alpha relative to the Ancestral Variant (Du et al. 2022; Campbell et al. 2021) We assumed that the rate of symptomatic disease was the same for all pre-Omicron variants, but that it was dramatically decreased for Omicron. (Yu et al. 2022) Rates of severe disease for Alpha and Delta relative to the Ancestral Variant were taken directly from the literature. (Fisman and Tuite 2021) Rates of severe disease for the Omicron Variant were only available in the literature relative to the Delta Variant, so we combined the odds ratios. (Nyberg et al. 2022)

| Variant | Incubation Period | $R_{0}$ (upper bound) | $\mathcal{B}_{j}^{\text{max}}$ | $OR_{\text{sev}}$ | $OR_{\text{symp}}$ |
| --- | --- | --- | --- | --- | --- |
| Ancestral | 6.5 | 5.0 | 5.7 | 1 (ref) | 1 (ref) |
| Alpha | 5.0 | 6.5 | 13.7 | 1 | 1.42 - 1.63 |
| Delta | 4.4 | 6.7 | 20.6 | 1 | 1.78 - 2.40 |
| Omicron | 3.4 | 6.7 | 50.2 | 0.2 - 0.3 | 0.69 - 1.03 |

Supplementary Table S4: The transitions used in the backcasting model. Almost all entries $Y_{i\to i^{'},j}^{I}=0$ and $Y_{i\to i^{'},j}^{V}=0$ except for those listed below, which are equal to one. Each row corresponds to a different value for i and the entries indicate the value of i' for which $Y_{i\to i^{'},j}^{I/V}=1$. The first two columns correspond to $Y^{I}$ with $j\in\{\text{anc},\text{alp},\text{del}\}$ and $j=\text{omi}$, respectively. The third and fourth columns correspond to $Y^{V}$with $j\in\{\text{pri, bst}\}$ and $j=\text{bvb}$, respectively.

| Class (i) | Pre-Omicron Infection | Omicron Infection | Primary/ Booster | Bivalent Booster |
| --- | --- | --- | --- | --- |
| Naïve | Natural Immunity | Natural Immunity (Omicron) | Vaccinated | - |
| Vaccinated | Hybrid Immunity | Hybrid Immunity (Omicron) | Boosted | Boosted (Bivalent) |
| Boosted | Boosted Hybrid Immunity | Boosted Hybrid Immunity (Omicron) | Boosted | Boosted (Bivalent) |
| Boosted (Bivalent) | Boosted Hybrid Immunity (Omicron) | Boosted Hybrid Immunity (Omicron) | Boosted (Bivalent) | Boosted (Bivalent) |
| Natural Immunity | Natural Immunity (Multiple) | Natural Immunity (Multiple, Omicron) | Hybrid Immunity | Hybrid Immunity (Omicron) |
| Natural Immunity (Multiple) | Natural Immunity (Multiple) | Natural Immunity (Multiple, Omicron) | Hybrid Immunity | Hybrid Immunity (Omicron) |
| Hybrid Immunity | Hybrid Immunity | Hybrid Immunity (Omicron) | Boosted Hybrid  Immunity | Boosted Hybrid Immunity (Omicron) |
| Boosted Hybrid Immunity | Boosted Hybrid Immunity | Boosted Hybrid Immunity (Omicron) | Boosted Hybrid  Immunity | Boosted Hybrid Immunity (Omicron) |
| Natural Immunity (Omicron) | Natural Immunity (Multiple, Omicron) | Natural Immunity (Multiple, Omicron) | Hybrid (Omicron) | Hybrid (Omicron) |
| Natural Immunity (Multiple, Omicron) | Natural Immunity (Multiple, Omicron) | Natural Immunity (Multiple, Omicron) | Hybrid (Multiple, Omicron) | Hybrid (Multiple, Omicron) |
| Hybrid Immunity (Omicron) | Hybrid Immunity (Omicron) | Hybrid Immunity (Omicron) | Boosted Hybrid Immunity (Omicron) | Boosted Hybrid Immunity (Omicron) |
| Boosted Hybrid Immunity (Omicron) | Boosted Hybrid Immunity (Omicron) | Boosted Hybrid Immunity (Omicron) | Boosted Hybrid Immunity (Omicron) | Boosted Hybrid Immunity (Omicron) |

Supplementary Table S5: Summary of parameters used in simulations

| Parameter | Description | Value | Source |
| --- | --- | --- | --- |
| $\lambda_{j}^{T}$ | Infection rate | $\text{Variable}$ | Calibrated to Hospitalization Data |
| $\nu_{j}^{T}$ | Vaccination rate | $\text{Variable}$ | Calibrated to Vaccination Data |
| $\eta_{j}^{T}$ | Transmission parameter | Variable | Calculated |
| $\omega_{V}$ | Immune waning rate (Vaccine) | $7.1*10^{-3}\text{day}^{-1}$ | Fixed |
| $\omega_{R}$ | Immune waning rate (Infection) | $7.1*10^{-3}\text{day}^{-1}$ | Fixed |
| $\omega_{P}$ | Immune waning rate (Antibodies, Roche) | $7.3*10^{-4}-1.6*10^{-3}\text{day}^{-1}$ | Calibrated to Torres Ortiz et al. (2022) |
| $\omega_{P}$ | Immune waning rate (Antibodies, Abbott) | $3.5*10^{-3}-5.9*10^{-3}\text{day}^{-1}$ | Calibrated to Torres Ortiz et al. (2022) |
| $C_{ij}^{V}$ | Relative susceptibility to infection | $\leq1\text{(Table 1 )}$ | Meta-regression |
| $M_{ij}^{V}$ | Efficacy against severe infections | $\leq1\text{(Table 1 )}$ | Meta-regression |
| $Y^{V}$ | Infection Transition | $0\text{ or }1\text{(Supp. Table S2 )}$ | Model Assumption |
| $Y^{I}$ | Vaccination Transition | $0\text{ or }1\text{(Supp. Table S2 )}$ | Model Assumption |
| $\mu_{\text{anc}\text{,1}}$ | Proportion severe infections (naive) | $0.001-0.003 \text{(0-17 years)}$ | Salje et al. (2020) |
| $\mu_{\text{anc}\text{,2}}$ | Proportion severe infections (naive) | $0.008-0.02 \text{(18-49 years)}$ | Salje et al. (2020) |
| $\mu_{\text{anc}\text{,3}}$ | Proportion severe infections (naive) | $0.021-0.054 \text{(50-64 years)}$ | Salje et al. (2020) |
| $\mu_{\text{anc}\text{,4}}$ | Proportion severe infections (naive) | $0.021-0.054 \text{(65+ years)}$ | Salje et al. (2020) |
| $B_{a^{'}\to a}$ | Contact rates per day | $\text{Table S3}$ | Prem, Cook, and Jit (2017) |

### Meta-regression of Efficacy Parameters

We used data from 1741 estimates of efficacy from 123 studies, which have been previously described in a systematic review and meta-regression, to estimate efficacy parameters. For the present study we used a simplified and modified version of the analysis, fitting mixed-effects linear models for protection against asymptomatic or mild infection $C$, and severe disease $M$ compared to naive individuals (i.e. unvaccinated with no history of infection). For each outcome, we fit two separate models depending on whether the infecting variant “matched” the pre-existing immunity, yielding a total of four models. Vaccination, boosting or prior infection with a pre-Omicron variant was assumed to confer matched immunity to all pre-Omicron variants. Similarly, any Omicron infection was assumed to confer “matched” immunity to any Omicron reinfection. Other situations were categorized as “unmatched”. Within each model, we consider five types of immunity: primary vaccination, booster doses, prior infection, hybrid immunity, and boosted hybrid immunity. We use a multi-level structure to predict the $n$th estimate from the $m$th study ($C_{nm}$ or $M_{nm}$) based on 1) the source of immunity ($X_{nm}$) which effects both initial immunity $\boldsymbol{\beta}_{0}$ and the change in immunity over time $\boldsymbol{\beta}_{1}$, 2) the time since immune conferring event ($t_{nm}$), 3) study-level random effects on initial immunity ($u_{m,0}$) and change in immunity ($u_{m,1}$), 4) corresponding estimate level random effects ($v_{nm,0}$ and $v_{nm,1}$), and 5) an estimate specific residual $\epsilon_{nm}$ distributed according to the study-reported standard deviation $\sigma_{nm}$.

For $C$ the model equations are

$$\begin{matrix} C_{nm} & =\boldsymbol{\beta}_{0}\mathbf{X}_{nm}+u_{m,0}+v_{nm,0}+\left( \boldsymbol{\beta}_{1}\mathbf{X}_{nm}+u_{m,1}+v_{nm,1} \right)\left( 1-exp\left( -\omega t_{nm} \right) \right)+\epsilon_{nm} \\ \left( \begin{matrix} u_{0,m} \\ u_{1,m} \\ \end{matrix} \right) & \mathcal{\sim N}\left( 0,\Sigma_{u} \right) \left( \begin{matrix} v_{0,nm} \\ v_{1,nm} \end{matrix} \right)\mathcal{\sim N}\left( 0,\Sigma_{v} \right) \epsilon_{nm}\mathcal{\sim N}\left( 0,\sigma_{nm}^{2} \right) \end{matrix}$$

In the above $\omega=0.05/\text{week}$, represents time-scale of immune loss. We use an equivalent setup for estimates of $M$

For the sake of model parsimony and biological plausibility, we grouped together several model parameters, i.e. components of $\boldsymbol{\beta}_{0}$ and $\boldsymbol{\beta}_{1,}$ to be identical (see [Supplementary Table S2](#tab:Efficacy-Parameter-Table)). The bivalent booster was assumed to confer “matched” immunity to all variants. Models were fit in R using the metafor package version 4.8.

### Durability of Seropositivity

We modeled the decay of anti-N seropositivity following infection as a two-step process, $R\to P\to W$ where $R$, $P$, and $W$ are immune subclasses as defined in [Supplementary Table S1](#tab:define-subclasses). The proportion of individuals in each subclass, following infections, obeys the following system of differential equations.

$$\begin{matrix} \frac{dR}{dt} & =-\omega_{R}R \\ \frac{dP}{dt} & =\omega_{R}R-\omega_{P}P \\ \frac{dW}{dt} & =\omega_{P}P \\ R\left( 0 \right) & =1 P\left( 0 \right)=W\left( 0 \right)=0 \end{matrix}$$

The proportion of seropositive individuals at time $t$ is $R\left( t \right)+P\left( t \right)$. We fit the values for $\omega_{R}$ and $\omega_{P}$ to clinical data ([Supplementary Figure S1](#fig:Sero-positivity-duration)), deriving separate parameterizations for Abbott-N and Roche-N assays.(Torres Ortiz et al. 2022)

In our full model, the proportion of seropositive individuals is

$$\text{Fraction seropositive}=\frac{\sum_{i} \left( X_{i}^{R}+X_{i}^{P} \right)}{X_{0}+\sum_{i} \left( X_{i}^{R}+X_{i}^{P}+X_{i}^{W}+X_{i}^{V} \right)}$$

As a validation of our methodology, we compared this modeled seropositivity with serosurveillance data from the CDC’s covid tracker ([Supplementary Figure S2](#fig:Sero-comparison)).(Disease Control and Prevention 2023)


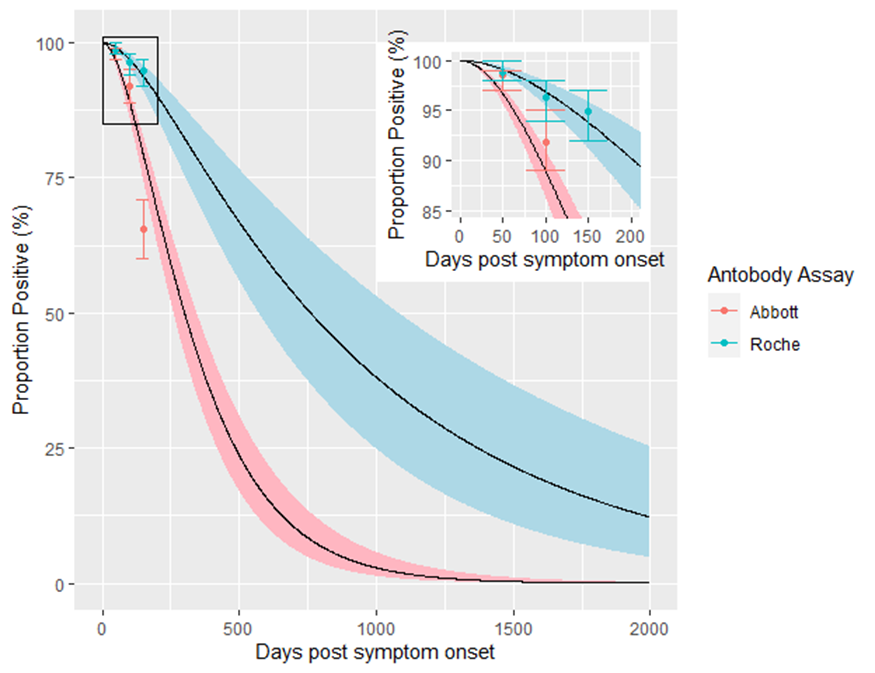


Figure S1: Decay in seropositivity over time in the Abbott and Roche antibody assays. Ribbons show the dynamics of the model; points show data collected from Torres Ortiz et al. (2022).


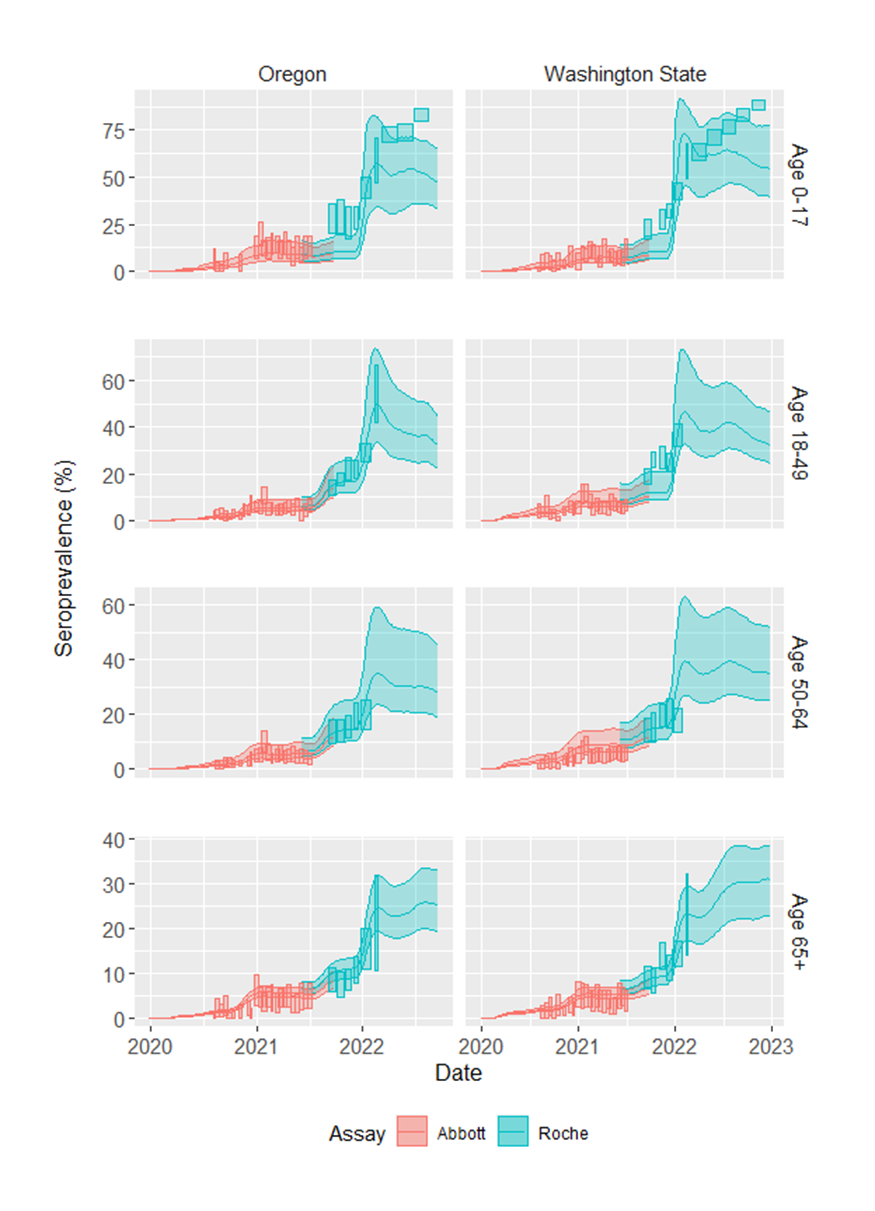


Figure S2: Comparison of model predicted seropositivity for anti-nucleocapsid antibodies with observation. All individuals in either the P or R subclasses were by definition seropositive. We ran two versions of the model reflecting the properties of either the Abbott or Roche assay. Ribbons show model prediction with 95% credible interval. Rectangles indicate the timing state-wide of seroprevalence surveys and the 95% confidence interval estimated for each.

### Calculation of Population Level Protection

Throughout the simulation we tracked the population-level protection against infection with variant $j$, $EC_{j}^{T}$, and severe injections with variant $j$, $EM_{j}^{T}$. This is defined as the reduction in each outcome relative to what would be observed in a naive population during the time period $[t_{T},t_{T+1})$. We decomposed this protection into contributions from different forms of immunity, defining the contribution from immunity class $i$ and subclass $k$ as $EC_{j}^{ik}$ and $EM_{j}^{ik}$. We defined the protection against the prevalent variant(s) as the average of the protection against individual variants weighted by the current incidence $\lambda_{j}^{T}$.

$$\begin{matrix} \text{Total population} N & =\sum_{i} \sum_{k\in\{V,R,P,W\}} X_{i}^{k}\left( t \right) \\ \text{Effective immunity against infection} EC_{j} & =1-\frac{\sum_{i} \sum_{k\in\{V,R,P,W\}} \int_{t_{T}}^{t_{T+1}} C_{ij}^{k}X_{i}^{k} dt}{N\left( t_{T+1}-t_{T} \right)} \\ & =\sum_{i} \sum_{k\in\{V,R,P,W\}} EC_{ij}^{k} \\ \text{Effective immunity against severe infection} EM_{j} & =1-\frac{\sum_{i} \sum_{k\in\{V,R,P,W\}} \int_{t_{T}}^{t_{T+1}} C_{ij}^{k}M_{ij}^{k}X_{i}^{k} dt}{N\left( t_{T+1}-t_{T} \right)} \\ & =\sum_{i} \sum_{k\in\{V,R,P,W\}} EM_{ij}^{k} \\ \text{Contribution from to class i, subclass j} EC_{ij}^{k} & =\frac{\int_{t_{T}}^{t_{T+1}} \left( 1-C_{ij}^{k} \right)X_{i}^{k} dt}{N\left( t_{T+1}-t_{T} \right)} \\ EM_{ij}^{k} & =\frac{\int_{t_{T}}^{t_{T+1}} \left( 1-C_{ij}^{k}M_{ij}^{k} \right)X_{i}^{k} dt}{N\left( t_{T+1}-t_{T} \right)} \\ \text{Effective immunity to prevalent }\text{variant}\text{(s)} EC & =\sum_{j} \lambda_{j}^{T}EC_{j}/\sum_{j} \lambda_{j}^{T} \\ EM & =\sum_{j} \lambda_{j}^{T}EM_{j}/\sum_{j} \lambda_{j}^{T} \\ EC_{i}^{k} & =\sum_{j} \lambda_{j}^{T}EC_{ij}^{k}/\sum_{j} \lambda_{j}^{T} \\ EM_{i}^{k} & =\sum_{j} \lambda_{j}^{T}EM_{ij}^{k}/\sum_{j} \lambda_{j}^{T} \end{matrix}$$

### Estimation of $\lambda$ and $\nu$

We used Markov Chain Monte Carlo (MCMC) to estimate the force parameters $\lambda_{j}^{T}$ and $\nu_{j}^{T}$. Given the high-dimensional nature of this problem, to speed convergence we used an expectation-maximization method to propose new steps in the chain rather than sampling from a gaussian kernel.

The expected number of vaccinations by type and the expected number of severe infections by variant, each week, $\mathbf{Z}=\{Z_{j}^{T};\forall T,j\}$, is a product of the forces of vaccination and infection, $\boldsymbol{\theta}=\{\nu_{j}^{T},\lambda_{j}^{T};\forall T,j\}$, with an effective population size, $\mathbf{P}=\{P_{j}^{T};\forall T,j\}$. This effective population size is a latent hidden variable, which can be derived from our system of ODEs as a function of $\mathbf{P}=\mathbf{F}\left( \boldsymbol{\theta} \right)$.

$$P_{j}^{T}=\left\{ \begin{matrix} \int_{t_{T}}^{t_{T+1}} \sum_{i} \sum_{k\in\{S,A,W\}} E_{ij}^{k}X_{i}^{k}\left( t \right) dt & j\in\{\text{pri},\text{bst},\text{bvb}\} \\ \int_{t_{T}}^{t_{T+1}} \sum_{i} \sum_{k\in\{V,S,A,W\}} C_{ij}^{k}M_{ij}^{k}SEV_{ij}^{k}X_{i}^{k}\left( t \right) dt & j\in\{\text{anc},\text{alp},\text{del},\text{omi}\} \end{matrix} \right.$$

An initial guess, $\boldsymbol{\theta}^{\left( 0 \right)}$, was obtained by assuming that the entire population of $P_{0}$ individuals was eligible for vaccination and had rates of severe disease equal to that of the naive population. These initial guesses were overestimates as the effective population size shrank over time.

$$\theta_{j}^{T,\left( 0 \right)}=\left\{ \begin{matrix} \hat{Z}_{j}^{T}/P_{0} & j\in\{\text{pri},\text{bst},\text{bvb}\} \\ \hat{Z}_{j}^{T}/\left( M_{i=\text{Naive},j}SEV_{i=\text{Naive},j}P_{0} \right) & j\in\{\text{anc},\text{alp},\text{del},\text{omi}\} \end{matrix} \right.$$

At each iteration $n=1,\ldots,N$ the following steps were performed

1. We updated the susceptible population via $\mathbf{P}^{\left( n \right)}=\mathbf{F}\left( \boldsymbol{\theta}^{\left( n \right)} \right)$.

1. We calculated the likelihood, $L^{\left( n \right)}=G\left( \hat{\mathbf{Z}};\boldsymbol{\theta}^{\left( n \right)},\mathbf{P}^{\left( n \right)} \right)$, using the observed data $\hat{\mathbf{Z}}$ assuming a poisson distribution.

$$\text{Likelihood} G\left( \hat{\mathbf{Z}};\boldsymbol{\theta},\mathbf{P} \right):=\prod_{j,T} \frac{e^{-\theta_{j}^{T}P_{j}^{T}}}{\hat{Z}_{j}^{T}!}\left( \theta_{j}^{T}P_{j}^{T} \right)^{\hat{Z}_{j}^{T}}$$

2. We proposed a new parameter set, ${\tilde{\boldsymbol{\theta}}}^{\left( k \right)}$ using the gamma-distributed conjugate prior of the above likelihood. Using this proposed parameter set, we calculated the corresponding adjusted population ${\tilde{\mathbf{P}}}^{\left( k \right)}=\mathbf{F}\left( {\tilde{\boldsymbol{\theta}}}^{\left( k \right)} \right)$ and proposed log-likelihood is $\tilde{L}^{\left( k \right)}=G\left( \mathbf{Z},{\tilde{\boldsymbol{\theta}}}^{\left( k \right)},{\tilde{\mathbf{P}}}^{\left( k \right)} \right)$.

$$\tilde{\theta}_{j}^{T}\sim\text{Gamma}\left( \text{shape}=\hat{Z}_{j}^{T},\text{rate}=P_{j}^{T} \right)$$

3. For iterations $n=1,\ldots N/2$, the new parameter set was either accepted or rejected with probability $A_{1}$, according to the likelihood-ratio. This ensures rapid convergence towards the most likely parameter fits.

$$A_{1}\left( \boldsymbol{\theta}^{\left( k \right)},{\tilde{\boldsymbol{\theta}}}^{\left( k \right)} \right)=min\left( 1,\frac{\tilde{L}^{\left( k \right)}}{L^{\left( k \right)}} \right)$$

- For iterations $n=N/2+1,\ldots N$, the new parameter set was accepted with probability $A_{2}$, accounting for the differences in the probability of the forward and backwards steps. This prevents over sampling of the highest likelihood region of parameter space.

$$A_{2}\left( \boldsymbol{\theta}^{\left( k \right)},{\tilde{\boldsymbol{\theta}}}^{\left( k \right)} \right)=min\left( 1,\frac{\tilde{L}^{\left( k \right)}\text{Prob}\left( {\tilde{\boldsymbol{\theta}}}^{\left( k \right)}\to\boldsymbol{\theta}^{\left( k \right)} \right)}{L^{\left( k \right)}\text{Prob}\left( \boldsymbol{\theta}^{\left( k \right)}\to{\tilde{\boldsymbol{\theta}}}^{\left( k \right)} \right)} \right)$$

$$\text{Prob}\left( \boldsymbol{\theta}^{\left( k \right)}\to{\tilde{\boldsymbol{\theta}}}^{\left( k \right)} \right) H\left( \tilde{\boldsymbol{\theta}};\hat{\mathbf{Z}},\mathbf{F}\left( \boldsymbol{\theta} \right) \right):=\prod_{j,T} \frac{e^{-\tilde{\theta}_{j}^{T}P_{j}^{T}}}{\tilde{\theta}_{j}^{T}\Gamma\left( \hat{Z}_{j}^{T} \right)}\left( \tilde{\theta}_{j}^{T}P_{j}^{T} \right)^{\hat{Z}_{j}^{T}}$$

### Calculation of Transmission.

In time interval *T*, the total number of infections in age group $a$, $I_{j,a}^{T}$, is the product of the force of infection $\lambda_{j,a}^{T}$, the population size $N_{a}$ and the population-level relative susceptibility $1-EC_{j,a}^{T}$, and the length of the interval $t_{T+1}-t_{T}$. The force of infection is a combination of “exogenous” infections which originate from outside the region and “endogenous” infections originating from within the region. As we used a time interval of one week, we assumed that the endogenous force of infection depended on 1) the number of infections in the previous week in all age groups $a'$ $I_{j,a'}^{T-1}$, 2) the per-capita contact rate of each group $a'$ with age group $a$, $B_{a'\to a}$ (Supplementary Table S6), and 3) time, variant, and age specific parameter, $\eta_{j,a}^{T}$. The parameter $\eta_{j,a}^{T}$ is a composite of the transmissability of variant $j$ as well as changes in behavior such as social distancing, workplace or school closure, and masking, which varied over time and by age group and the effectiveness of which may differ by variant. We note that for early variants, the generation time corresponded to roughly one week, however for the Omicron Variant, one week was enough for multiple rounds of infection, this was reflected in potentially much higher values for $\eta$, as described below.

$$\begin{matrix} \begin{matrix} \text{All Infections in age group }a \\ j\in\{\text{anc},\text{alp},\text{del},\text{omi}\},\forall T \end{matrix} I_{j,a}^{T} & =\lambda_{j,a}^{T}N_{a}\left( 1-EC_{j,a}^{T} \right)\left( t_{T+1}-t_{T} \right) \\ \lambda_{j,a}^{T} & =\underset{\text{Exogenous Infection}}{\underset{⏟}{\lambda_{j,0}^{T}}}+\underset{\text{Endogenous Infection}}{\underset{⏟}{\eta_{j,a}^{T}\sum_{a'} B_{a'\to a}\eta_{j,a'}^{T}I_{j,a'}^{T-1}}} \end{matrix}$$

In a completely susceptible population (i.e $EC_{a}^{T}=0$) with no exogenous infection ($\lambda_{j,0}^{T}=0$) the number of infections from one week to the next initially evolved according to

$$I_{j,a}^{T}=N_{a}\eta_{j,a}^{T}\sum_{a'} B_{a'\to a}\eta_{j,a'}^{T}I_{j,a'}^{T-1}$$

which can be expressed in a matrix-vector form as $\mathbf{I}_{j}^{T}=\mathbf{B}_{j}^{T}\mathbf{I}_{j}^{T-1}$ using the time dependent contact matrix $\mathbf{B}_{j}^{T}=\text{diag}\left( \boldsymbol{\eta}_{j}^{T} \right)\mathbf{B} \text{diag}\left( \boldsymbol{\eta}_{j}^{T} \right)$ where $\mathbf{B}$ is the contact matrix in the absence of social distance (Supplementary Table S6).

The leading eigenvalue of $\mathbf{B}_{j}^{T}$, $\mathcal{B}_{j}^{T}$, represents the weekly growth rate from one week to the next. We constrained this value to be no greater than a variant-specific maximum value, $\mathcal{B}_{j}^{\text{max}}$, estimated from literature estimates of $R_{0}$ and incubation time for each variant (Supplementary Table S3).(Wu et al. 2022; Du et al. 2022) To estimate $\eta_{j,a}^{T}$ and $\lambda_{j,a,0}^{T}$ we solved the constrained non-linear system.

$$\begin{matrix} \eta_{j}^{T} & \text{minimizes} \sum_{a} \left( \lambda_{j,a}^{T}-\eta_{j,a}^{T}\sum_{a'} B_{a'\to a}\eta_{j,a'}^{T}I_{j,a'}^{T-1} \right)^{2} \\ 0 & \leq\eta_{j}^{T}<\eta_{j}^{\text{max}} \mathcal{B}\left( \boldsymbol{\eta}_{j}^{max} \right)=\mathcal{B}_{j}^{max} \\ \lambda_{j,a,0}^{T} & =\lambda_{j,a}^{T}-\eta_{j,a}^{T}\sum_{a'} B_{a'\to a}\eta_{j,a'}^{T}I_{j,a'}^{T-1} \end{matrix}$$

The quantities $\lambda_{j,a,0}^{T}$ and $\eta_{j,a}^{T}$ remain unchanged during our counterfactual scenarios. The estimated rates of exogenous and secondary endogenous infection by age group are shown in Supplementary Figures S7 and S8.

### Variant Specific Parameters

The rate of severe symptoms for variant $j$ in naive individuals, $\mu_{j}$, was defined as the product of the probability of developing symptoms $p_{\text{sev},j}$ multiplied by the probability of developing severe symptoms conditional on symptomatic disease $p_{\text{symp},j}$.

$$\mu_{j}=p_{\text{symp},j}p_{\text{sev},j}$$

For the Ancestral Variant, we set $\mu_{j}$ and $p_{\text{symp},j}$ using literature values estimated from prior modeling (Salje et al. 2020; Davies et al. 2020). We calculated $p_{\text{sev},j}$ by back-calculation. For infection with variant $j$ we applied odds ratios, $OR_{\text{symp},j}$ and $OR_{\text{sev},j}$, to the probability of developing symptoms and of symptomatic cases developing severe symptoms, respectively, due to the Ancestral Variant.

$$p_{\text{symp},j}=\frac{p_{\text{symp},\text{anc}}\times OR_{\text{symp},j}}{p_{\text{symp},\text{anc}}\times OR_{\text{symp},j}+\left( 1-p_{\text{symp},\text{anc}} \right)}$$

$$p_{\text{sev},j}=\frac{p_{\text{sev},\text{anc}}\times OR_{\text{sev},j}}{p_{\text{sev},\text{anc}}\times OR_{\text{sev},j}+\left( 1-p_{\text{sev},\text{anc}} \right)}$$

When back-calculating transmission week-by-week, we constrained our fits so that transmission never exceeds the highest values supported by the literature. Given a literature value for the basic reproductive number, $R_{0}$, and generation time (in days), then the weekly growth rate can be approximated as

$$\text{Max Weekly Growth Rate} \mathcal{B}_{j}^{\text{max}}=R_{0,j}^{\text{Generation Time}_{j}/7}$$

Given that the peak of transmissability coincides with the onset of symptoms, we approximate the generation time of each variant as being equal to the incubation time.

Supplementary Table S6: Contact network between age groups in the absence of social distancing. (Prem, Cook, and Jit 2017) Each row indicates the number of contacts an individual in a given age group would expect to have with members of the other age groups (matrix $\boldsymbol{B}$ with entries $B_{a'\to a})$. Values were rebalanced to match the King County population structure.

| Age Group | 0-17 | 18-49 | 50-64 | 65+ |
| --- | --- | --- | --- | --- |
| 0-17 | 12.36 | 5.30 | 1.68 | 1.01 |
| 18-49 | 2.54 | 9.85 | 2.43 | 0.65 |
| 50-64 | 2.07 | 6.24 | 3.41 | 0.89 |
| 65+ | 1.65 | 2.24 | 1.18 | 1.35 |

# References

Davies, Nicholas G, Petra Klepac, Yang Liu, Kiesha Prem, Mark Jit, and Rosalind M Eggo. 2020. “Age-Dependent Effects in the Transmission and Control of COVID-19 Epidemics.” *Nature Medicine* 26 (8): 1205–11.

Disease Control, Centers for, and Prevention. 2023. “COVID Data Tracker.” Department of Health; Human Services, CDC. <https://covid.cdc.gov/covid-data-tracker>.

Du, Zhanwei, Caifen Liu, Chunyu Wang, Lingfeng Xu, Mingda Xu, Lin Wang, Yuan Bai, et al. 2022. “Reproduction Numbers of Severe Acute Respiratory Syndrome Coronavirus 2 (SARS-CoV-2) Variants: A Systematic Review and Meta-Analysis.” *Clinical Infectious Diseases* 75 (1): e293–95.

Salje, Henrik, Cécile Tran Kiem, Noémie Lefrancq, Noémie Courtejoie, Paolo Bosetti, Juliette Paireau, Alessio Andronico, et al. 2020. “Estimating the Burden of SARS-CoV-2 in France.” *Science* 369 (6500): 208–11.

Torres Ortiz, Arturo, Fernanda Fenn Torrente, Adam Twigg, James Hatcher, Anja Saso, Tanya Lam, Marina Johnson, et al. 2022. “The Influence of Time on the Sensitivity of SARS-CoV-2 Serological Testing.” *Scientific Reports* 12 (1): 10517.

Wu, Yu, Liangyu Kang, Zirui Guo, Jue Liu, Min Liu, and Wannian Liang. 2022. “Incubation Period of COVID-19 Caused by Unique SARS-CoV-2 Strains: A Systematic Review and Meta-Analysis.” *JAMA Network Open* 5 (8): e2228008–8.

## Supplemental Results

### Calibration of Time-Varying Parameters


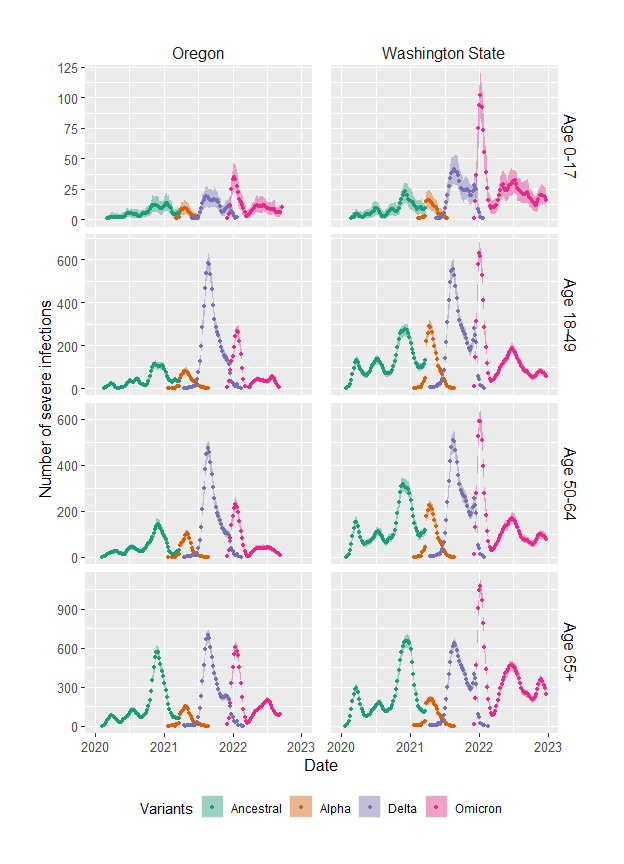


Supplementary Figure S3: Number of weekly infections resulting in severe outcomes (hospitalization and/or death with SARS-CoV-2 infection) in Oregon and Washington State over time. Points represent numbers estimated from deconvolution of case data from Washington Department of Health and Oregon health authority. Ribbon shows 95% credible interval of backcasting-model. Ribbon appears as a single line when variability is low.


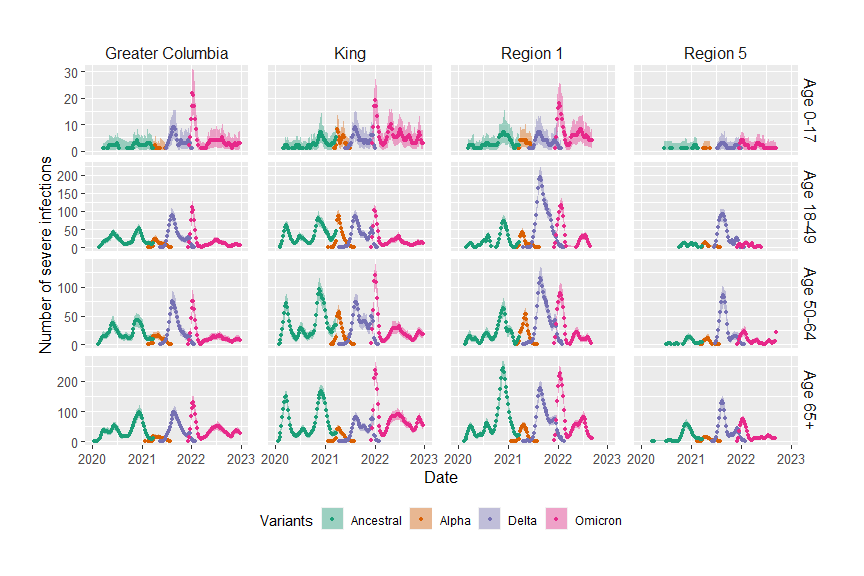


Supplementary Figure S4: Number of weekly infections resulting in severe outcomes (hospitalization and/or death with SARS-CoV-2 infection) in two regions each in Washington State and Oregon over time. Points represent numbers estimated from deconvolution of case data from Washington Department of Health and Oregon health authority. Ribbon shows 95% credible interval of backcasting-model. Ribbon appears as a single line when variability is low.


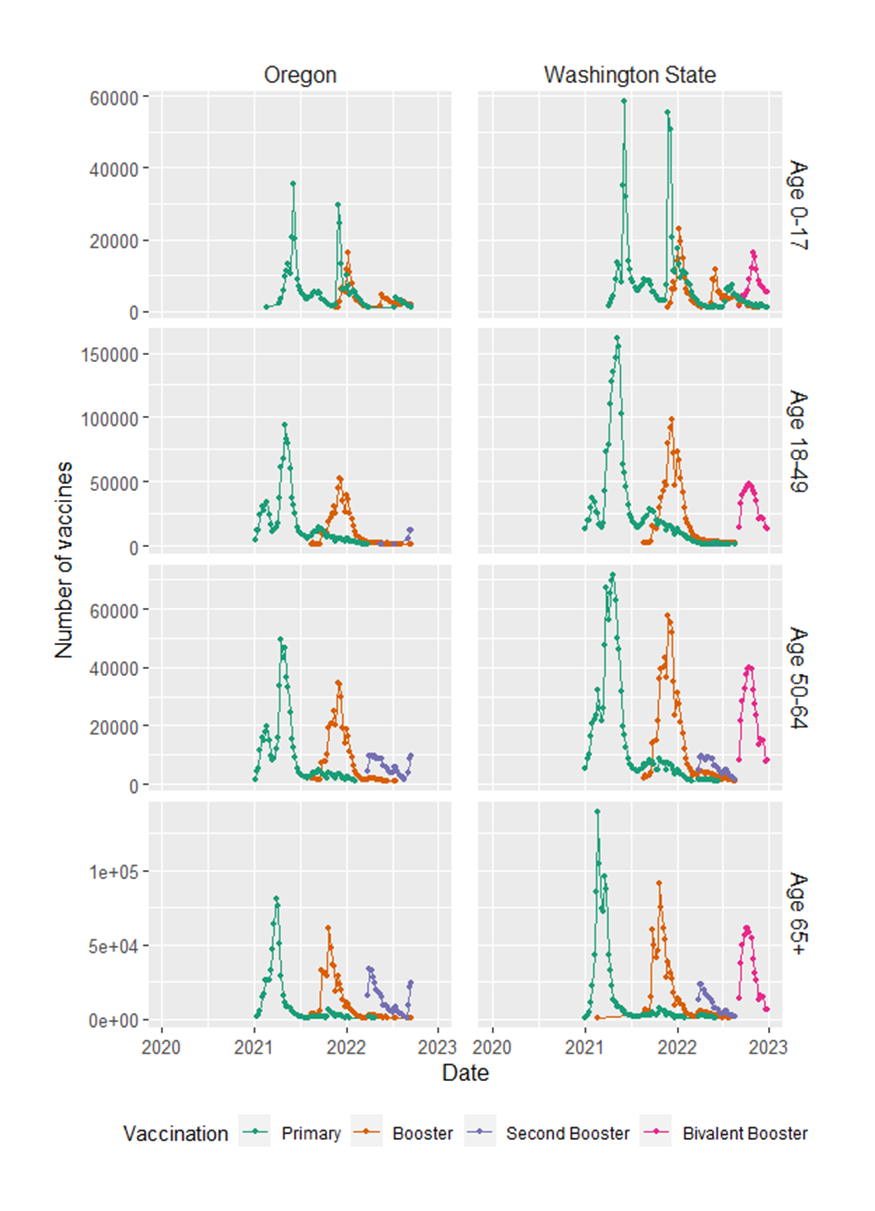


Supplementary Figure S5: Number of weekly vaccinations in Oregon and Washington State over time. Points represent numbers estimated from deconvolution of case data from Washington Department of Health and Oregon health authority. Ribbon shows 95% credible interval of backcasting-model. Ribbon appears as a single line when variability is low.


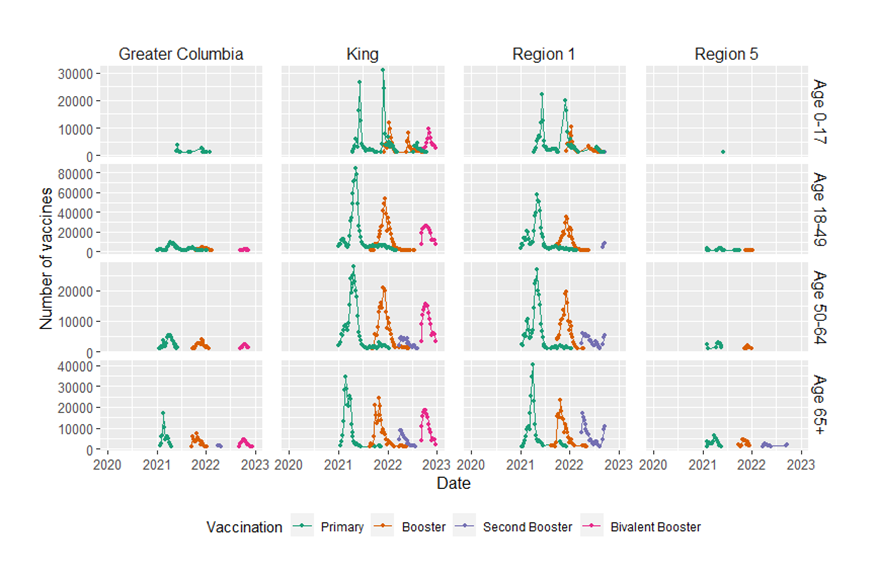


Supplementary Figure S6: Number of weekly vaccinations in two regions each of Washington State and Oregon over time. Points represent numbers estimated from deconvolution of case data from Washington Department of Health and Oregon health authority. Ribbon shows 95% credible interval of backcasting-model. Ribbon appears as a single line when variability is low.

### Transmission Parameters


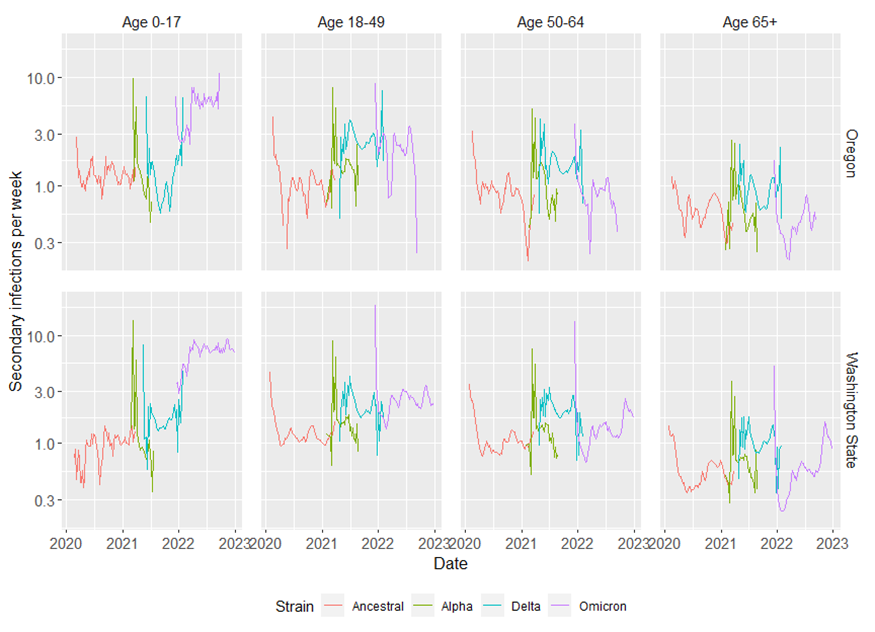


*Supplementary Figure S7: Number of new infections in a given week arising from a single index infection in the previous week assuming a completely naive population. Values were obtained from taking the row sums of the transmission matrix $\boldsymbol{B}_{j,a}^{T}$.*


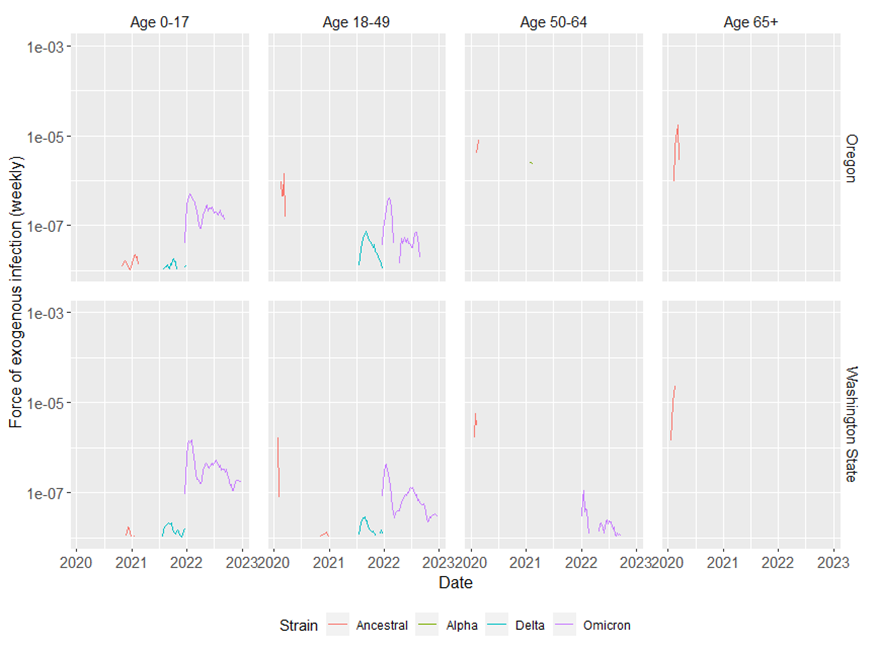


Supplementary Figure S8: Force of exogenous infection in Washington State and Oregon over time. Values of $\lambda_{j,a,0}^{T}$ over time for each variant. Note that if an age group has no exogenous infections for a given variant, then the infection was seeded from another age group.

### Population Level Protection


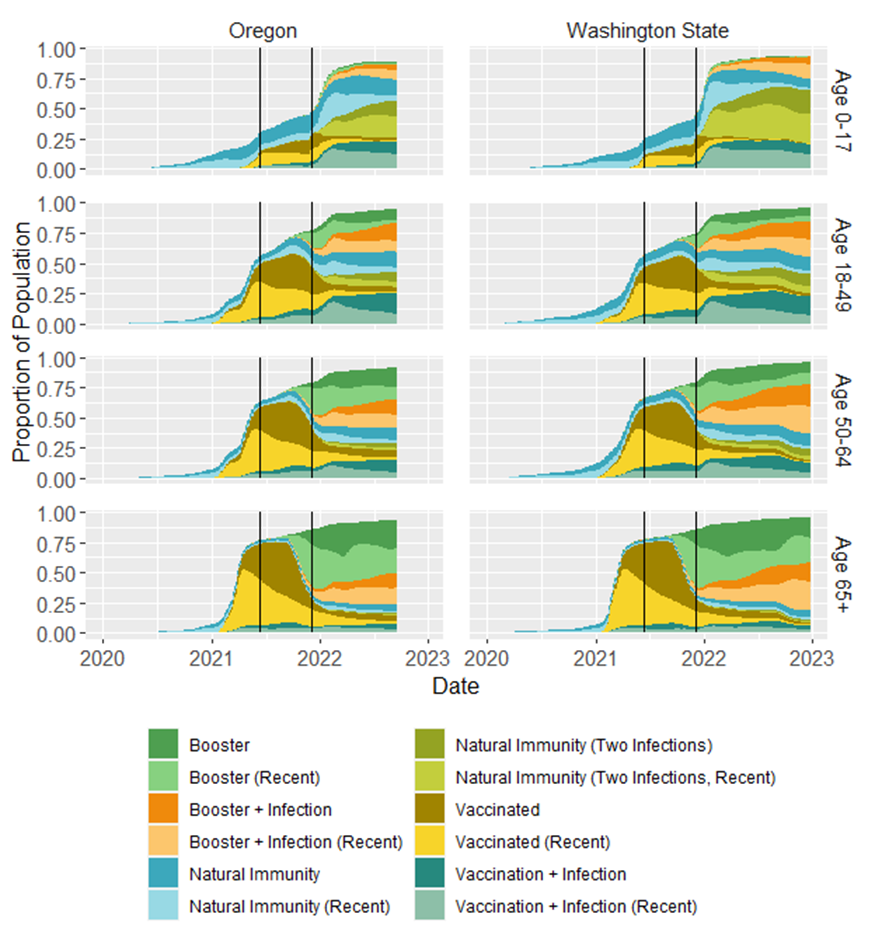


Supplementary Figure S9: Proportion of population in each immune class over time by age and state.


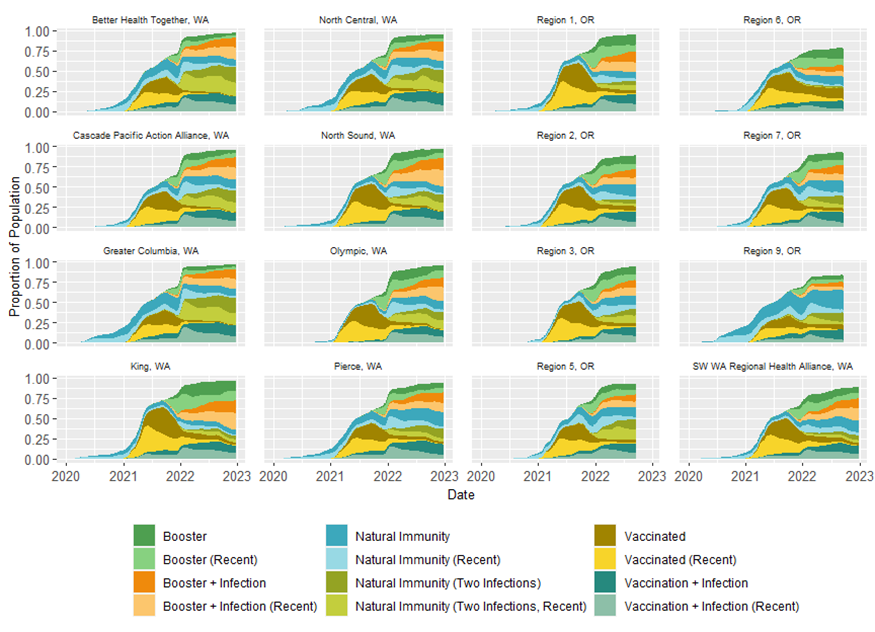


Supplementary Figure S10: Proportion of population in each immune class over time by region.


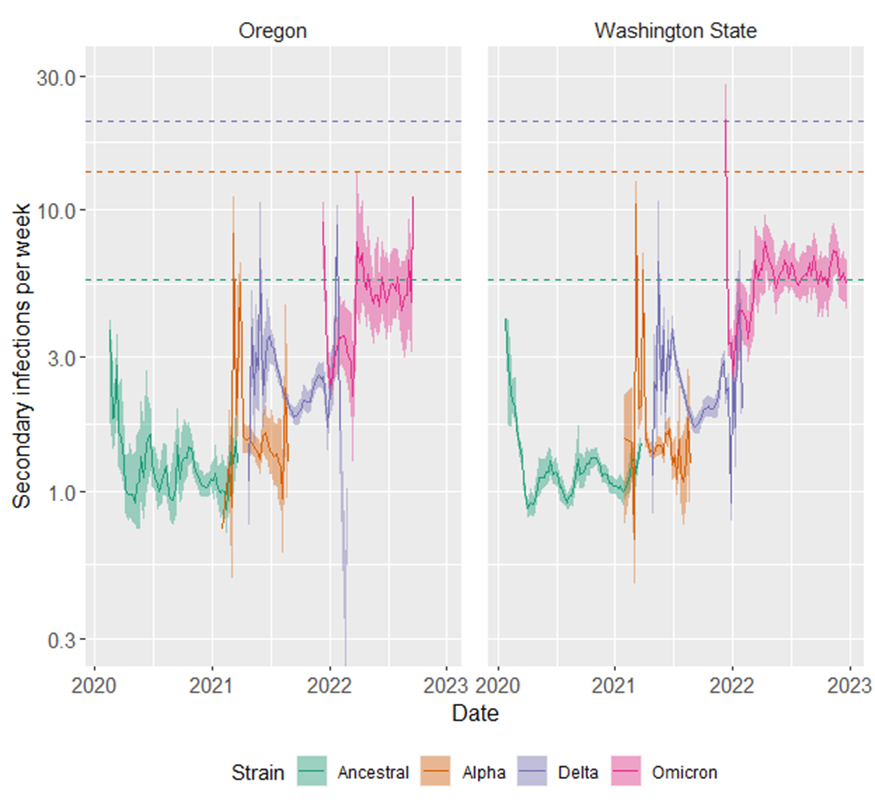


Supplementary Figure S11: Efficacy-adjusted weekly growth rate $\mathcal{B}_{j,r}^{T}$ for each variant $j$ and week $T$ over time in Oregon and Washington states. Lines and surrounding ribbons represent median and 95% credible interval. These numbers represent the ratio of new infections from one week to the next in a completely naive population. Dashed lines show upper bounds for weekly growth rates based on literature values for the basic reproductive numbers and serial intervals for each variant, $\mathcal{B}_{j}^{max}$ (value for Omicron is outside the vertical range of the plot).

### Counterfactual Infections


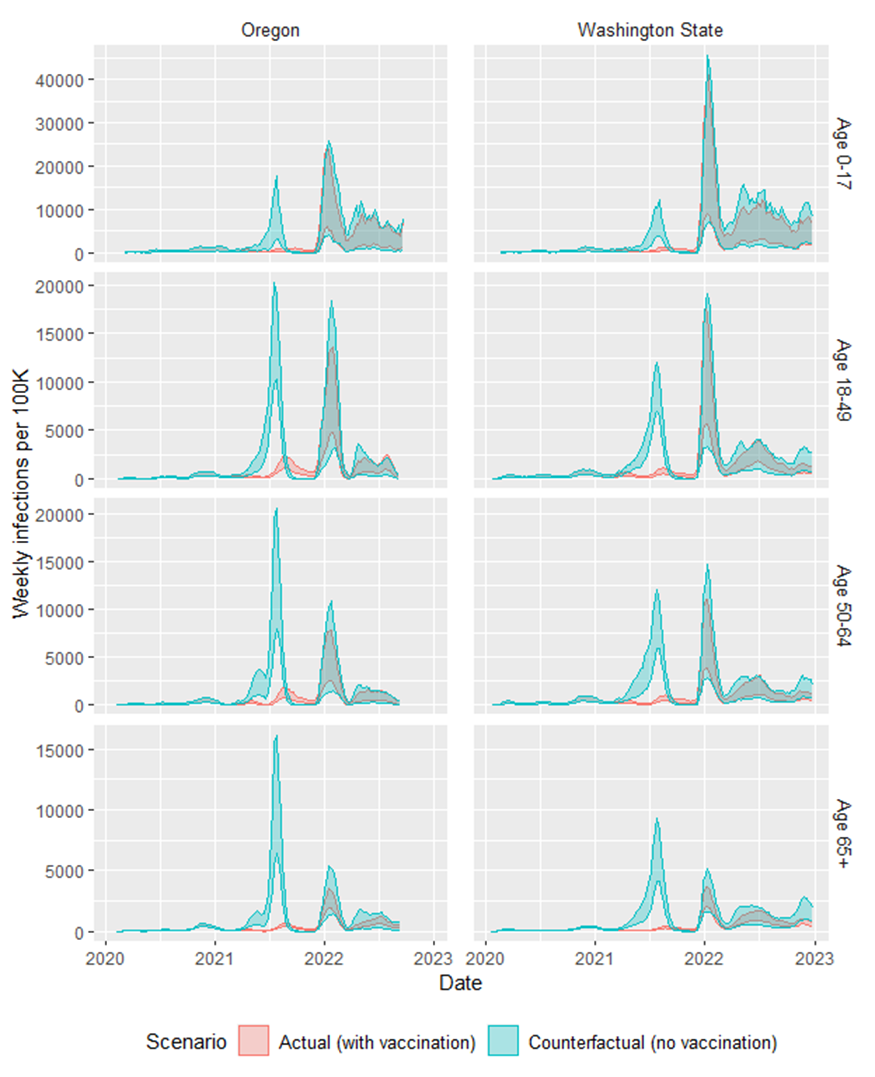


Supplementary Figure S12: Weekly numbers of SARS-CoV- 2 infections with and without vaccination in Washington and Oregon state. Severe infection is defined as any infection resulting in hospitalization or death. “Actual” scenario uses observed vaccination rates, “Counterfactual” scenario has no vaccination. All numbers are per 100K. Ribbon indicates 95% credible interval.

### No-booster Counterfactual


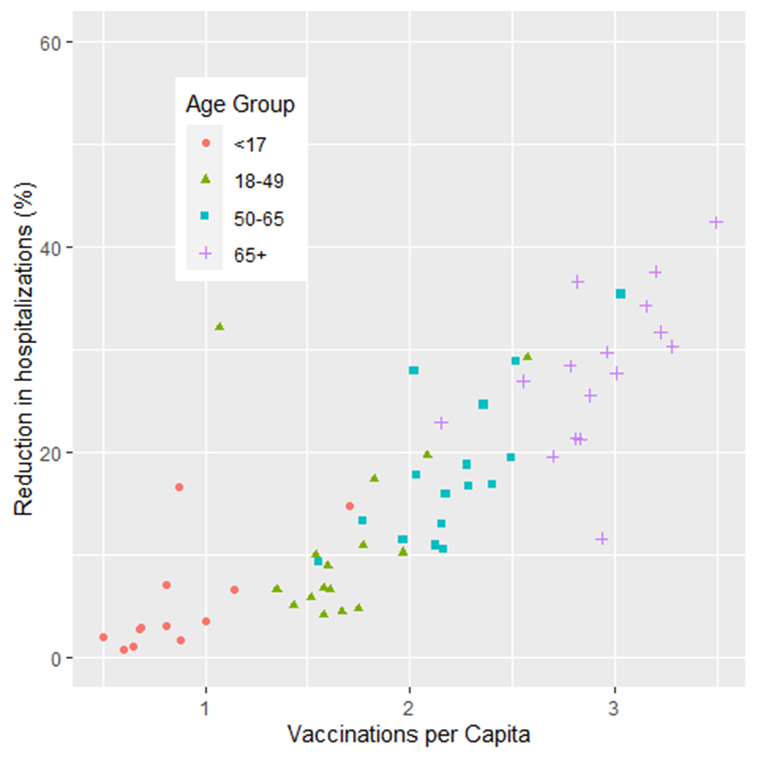


Supplementary Figure S13: Impact of booster doses on Omicron infection. Correspondence of vaccine uptake (in doses per person) and impact (as percent reduction in hospitalizations in simulation with boosters vs without). Each point corresponds to a single age group with a region of either Washington State and Oregon. Points represent medians across all simulations.
